# Mutational Landscapes and Phenotypic Spectrum of SWI/SNF-Related Intellectual Disability Disorders

**DOI:** 10.3389/fnmol.2018.00252

**Published:** 2018-08-03

**Authors:** Nina Bögershausen, Bernd Wollnik

**Affiliations:** Institute of Human Genetics, University Medical Center Göttingen, Göttingen, Germany

**Keywords:** Coffin-Siris syndrome, Nicolaides-Baraitser syndrome, SWI/SNF complex, BAF complex, SMARCA2, SMARCA4, ARID1B, ARID1A

## Abstract

Mutations in genes that encode proteins of the SWI/SNF complex, called BAF complex in mammals, cause a spectrum of disorders that ranges from syndromic intellectual disability to Coffin-Siris syndrome (CSS) to Nicolaides-Baraitser syndrome (NCBRS). While NCBRS is known to be a recognizable and restricted phenotype, caused by missense mutations in *SMARCA2*, the term CSS has been used lately for a more heterogeneous group of phenotypes that are caused by mutations in either of the genes *ARID1B, ARID1A, ARID2, SMARCA4, SMARCB1, SMARCE1, SOX11*, or *DPF2*. In this review, we summarize the current knowledge on the phenotypic traits and molecular causes of the above named conditions, consider the question whether a clinical distinction of the phenotypes is still adequate, and suggest the term “SWI/SNF-related intellectual disability disorders” (SSRIDDs). We will also outline important features to identify the *ARID1B*-related phenotype in the absence of classic CSS features, and discuss distinctive and overlapping features of the SSRIDD subtypes. Moreover, we will briefly review the function of the SWI/SNF complex in development and describe the mutational landscapes of the genes involved in SSRIDD.

## Introduction

The SWI/SNF (switch/sucrose non-fermenting) complex, first purified and characterized in yeast, is one of a group of ATP-dependent chromatin remodeling complexes, which regulate DNA accessibility at the nucleosome by mobilizing nucleosomes in an ATP-dependent manner (Kassabov et al., 2003; Zofall et al., 2006). This mechanism serves to establish an open chromatin structure, thereby enhancing DNA accessibility, and thus facilitating DNA transcription, replication, and repair. The ubiquitous expression of the complex components reflects their essential functions in development and thus also in human disease.

In the 1990s SWI/SNF complex components were recognized as tumor suppressor genes implicated in the carcinogenesis of pediatric tumors (Dunaief et al., 1994; Versteege et al., 1998). These early studies were followed by many next-generation-sequencing (NGS) studies in recent years, broadening the spectrum of human cancers associated with loss-of-function of different SWI/SNF components (Kadoch and Crabtree, 2015). Concurrently, and with the advent of NGS in the field of human genetics, the first mutations in chromatin-remodeling factors causing human developmental syndromes were identified. The groups of Hoyer et al. (2012), Santen et al. (2012), Tsurusaki et al. (2012), and Van Houdt et al. (2012) identified heterozygous, mainly *de novo*, mutations in the genes *ARID1B* (MIM: 614556), *ARID1A* (MIM: 603024), *SMARCA4* (BRG1 [MIM: 603254]), *SMARCB1* (SNF5 [MIM: 601607]), and *SMARCE1* (MIM: 603111) as the causes of syndromic intellectual disability (ID) and Coffin-Siris syndrome (CSS), and mutations in *SMARCA2* (MIM: 600014) as the cause of Nicolaides-Baraitser syndrome (NCBRS). Their results were confirmed by several groups (Wolff et al., 2012; Santen et al., 2013; Wieczorek et al., 2013; Sousa et al., 2014) and complemented by the description of heterozygous mutations in *SOX11* (MIM: 600898) (Tsurusaki et al., 2014a; Hempel et al., 2016) and in *ARID2* (MIM: 609539) in patients with CSS-like phenotypes (Bramswig et al., 2017). Most recently, truncating as well as missense heterozygous mutations in the SWI/SNF complex subunit gene *DPF2* (double plant homeodomain finger 2; MIM: 601671) were added to the list of genetic causes of CSS (Vasileiou et al., 2018).

With the detection of mutations in the SWI/SNF complex genes in patients with CSS, NCBRS, and overlapping phenotypes, the question has emerged whether these clinical entities represent separate and specific conditions or whether they should all be regarded as manifestations of one clinical spectrum. Some authors have claimed that NCBRS is a specific entity (Sousa et al., 2014) that is distinct from CSS, while others have noted that the clinical distinction can be quite difficult (Santen et al., 2013). We will show that the conditions caused by mutations in the SWI/SNF complex genes all belong to a clinical continuum with syndromic ID and mild CSS at the one end, more severe and atypical phenotypes in the middle, and NCBRS at the other end. To satisfy the need for a descriptive name for this spectrum, we suggest the term “SWI/SNF-related intellectual disability disorders (SSRIDDs)”.

In this review, we will summarize the clinical presentation of the conditions contained in the SSRIDD spectrum, discuss overlapping and distinctive features, and depict the mutational landscapes of the associated genes.

## The SWI/SNF Complex in Development

**FIGURE 1:**
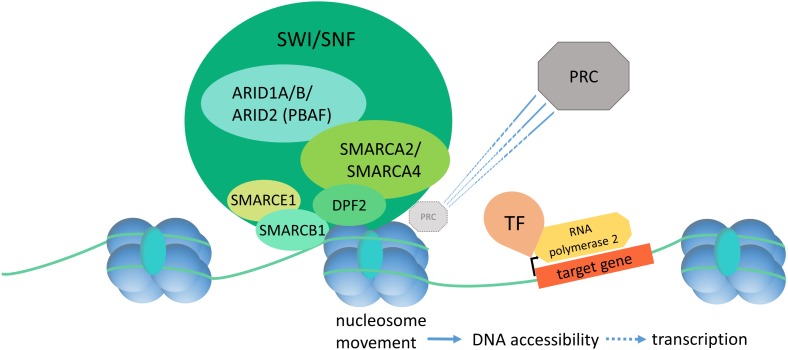
Diagram of SWI/SNF dependent chromatin remodeling. The SWI/SNF complex binds to DNA and histones and effectuates nucleosome displacement in order to enhance DNA accessibility and thereby initiation of the transcription machinery. SWI/SNF has a direct, SMARCA4-dependent PRC eviction capacity. PRC, polycomb repressive complex; TF, transcription factor.

Gene expression is closely linked to chromatin state and DNA accessibility for the transcriptional machinery. The term “chromatin remodeling” designates dynamic changes in chromatin state (closed vs. open, or hetero- vs. euchromatin) in response to extra- and intracellular cues. The dynamic regulation of chromatin state allows for orchestrated gene expression patterns as drivers of development and cell homeostasis. ATP-dependent chromatin remodeling complexes are the main actors in this process and can be divided into the Trithorax group (TrxG) and the Polycomb group (PcG) [reviewed in Schuettengruber et al. (2017)]. The SWI/SNF complex, as part of the TrxG, establishes a euchromatin state and promotes gene expression by increasing DNA accessibility (**Figure 1**). DNA accessibility is achieved by repositioning of nucleosomes, causing a loop of accessible DNA between the nucleosome entry and exit sites. TrxG complexes directly counteract the PcG complexes, which are responsible for chromatin compaction. The SWI/SNF complex has been shown to act in a direct and dynamic competition with the polycomb repressive complex 1 (PRC1) by a SMARCA4 dependent PRC1 eviction activity and it may serve as a “switch” to turn on chromatin decompaction, and thereby gene expression (Stanton et al., 2017). Consequently, a loss of SWI/SNF action, due to mutations in *SMARCA4*, causes polycomb accumulation and reduced chromatin accessibility at enhancer sites (Hodges et al., 2018), aberrant chromatin structure, and aberrant mitoses with formation of micronuclei (Bourgo et al., 2009).

In mammals, two differentially composed SWI/SNF complexes exist, also called BAF and PBAF. ARID1A/B are exchangeable core components of BAF and ARID2 is a core component of PBAF (Kadoch and Crabtree, 2015). SMARCA2 (BRM) and SMARCA4 (BRG1) are the mammalian homologs of yeast SWI and SNF. Either one can be found as the catalytic subunit of the two mammalian complexes, BAF and PBAF, which each contain up to 15 additional subunits, some shared and some distinctive (Schuettengruber et al., 2017). Together, the mammalian SWI/SNF complexes are composed of the products of at least 29 genes (Kadoch et al., 2017). This multitude of subunits allows for considerable diversity in complex composition, reflecting the multiple functions of the complexes during development and in the cellular homeostasis of highly ordered organisms.

Some of the BAF/PBAF subunits are tissue specific and it is an emerging concept that the combinatorial assembly of tissue-specific complexes is an important driver of lineage acquisition. For example, ACTL6B (BAF53b), DPF1 (BAF45b), and CREST are specific to post-mitotic neurons, whereas they are absent from neural progenitors (Kadoch et al., 2017). Toto et al. (2016) showed that the induction of two SWI/SNF components which are not expressed in embryonic stem cells, the catalytic subunit SMARCA2 (BRM) and the structural component SMARCD3 (BAF60C), coincides with the expansion of the transcriptional repertoire in muscle progenitors. These results suggest that the timely expression of certain SWI/SNF components directs stem cell fate in neurogenesis as well as in skeletal myogenesis. Recently, Smarcb1 has been shown to interact with p300 to facilitate acetylation and transcription factor binding at lineage-specific enhancer sites in murine embryonic fibroblasts (Alver et al., 2017), and depletion of Smarcb1 and Smarca4 led to a loss of expression of genes associated with tissue differentiation. These results place SWI/SNF at the top of a transcriptional cascade necessary for embryogenesis. It was also shown that inactivation of Smarcb1 leads to increased levels of p53, apoptosis, polyploidy, and growth arrest (Isakoff et al., 2005), indicating an essential role in cell cycle control. Studies in mouse and zebrafish have implicated SWI/SNF in embryonic development in general, and have shown specific roles in the development of the brain, the eye, the vascular system, and the heart (Supplementary Table S1).

*In summary*, as master regulators of chromatin structure and DNA transcription, the SWI/SNF complexes regulate cell cycle progression, cell fate acquisition and differentiation in a time- and tissue-specific manner.

## SWI/SNF-Related Intellectual Disability Disorders

**FIGURE 2:**
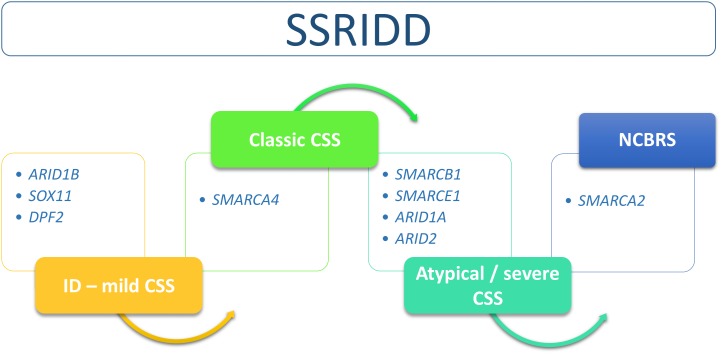
Flow chart of SSRIDD phenotypic spectrum ranging from syndromic ID over classic and atypical/severe CSS to NCBRS. Clinical categories within the spectrum are associated with specific SWI/SNF-associated genes.

The SSRIDDs comprise a spectrum that includes the classic CSS and NCBRS phenotypes and many overlapping syndromic conditions that range from syndromic ID to severe atypical CSS with strong NCBRS overlap (**Figure 2**).

The main clinical features of CSS are defined as ID, hypotonia, feeding problems, hypertrichosis, sparse scalp hair, thick eyebrows, long eyelashes, visual problems, thick alae nasi, large mouth, thick lower vermillion, malformed ears, lax joints, short fifth finger, and one or more underdeveloped nails, typically of the fifth finger (Coffin and Siris, 1970; Santen et al., 2013). The cardinal features of NCBRS are defined as ID, short stature, microcephaly, typical face, sparse hair, brachydactyly, prominent interphalangeal joints, behavioral problems, and seizures (Nicolaides and Baraitser, 1993; Sousa et al., 2009).

### Syndromic ID and CSS Caused by *ARID1B* Mutations

Two groups reported in 2012 almost concomitantly on the identification of heterozygous mutations in *ARID1B* as the cause of syndromic ID and CSS: Hoyer et al. (2012) and Santen et al. (2012). Since then it has become apparent that, firstly, *ARID1B* is the most frequently mutated gene in ID (Wright et al., 2018) and that, secondly, the phenotypic spectrum caused by mutations in this gene is very wide. Depending on the method of patient recruitment, there is remarkable ascertainment bias in the reported studies, which makes it difficult to ascertain the *ARID1B*-related phenotype.

Hoyer et al. (2012) described *de novo* mutations in *ARID1B* in patients with unexplained ID. Based on the finding of a heterozygous 2.5 Mb deletion, they performed a candidate gene study on 887 patients with unexplained ID and found eight further mutations (0.9%). The patients in this study already showed the phenotypic variability, which has since been broadly recognized. Patients had mild to severe ID, short to normal stature, all had psychomotor delay of variable degrees, three patients had seizures, one had hearing loss, one had a heart defect (atrial septal defect), all had at least three dysmorphic features, but there was no principal symptom other than ID that would lead the way in diagnosis. Six of nine patients had some abnormality of the hands and feet but only two of them had hypoplastic nails, only one of them specifically of the fifth toe. Most of the patients had an abnormal head shape and low-set, posteriorly rotated, and abnormally shaped ears. The facial aspect in most of these patients was rather coarse and some had sparse hair. Abnormalities on cerebral MRI were reported for three patients, however no agenesis of the corpus callosum (ACC) was observed.

Reports of ID with ACC caused by deletions or translocations resulting in loss of *ARID1B* had previously been published by Nagamani et al. (2009), Backx et al. (2011), and Halgren et al. (2012). Nagamani et al. (2009) observed ACC in two out of four patients, all of whom also showed microcephaly, developmental delay, dysmorphic features, and hearing loss. Halgren et al. (2012) described eight patients, harboring interstitial deletions or translocations (one patient) disrupting the *ARID1B* locus. All had ID and speech delay and four out of five examined with MRI had an abnormality of the corpus callosum. Halgren et al. also noticed autism or autistic traits in five of their patients.

The group of Santen et al. (2012), who described *de novo* mutations in *ARID1B* as the cause of CSS, took a different approach: they performed exome sequencing in three patients with a diagnosis of CSS. Naturally, the phenotype of these patients was much more concise. All patients showed a coarse facial aspect, thick eyebrows and broad nasal tips. They had moderate to severe ID and severe speech delay. Two out of three presented with brachydactyly 5 and/or a hypoplastic fingernail, and two had hypoplastic phalanges of the feet. All three had partial or full ACC. Santen et al. (2014) also identified three patients with deletions encompassing *ARID1B* by array CGH. These individuals also had moderate to severe ID and severely delayed to no speech as well as some overlapping facial features, but they were lacking the CSS cardinal symptom of hypoplastic fifth finger-/toenails. It was suggested that a diagnosis of CSS should be considered in all individuals with ID and speech impairment, particularly in the presence of ACC.

In a well-defined clinical cohort of SSRIDD patients with clinical diagnoses of CSS as well as NCBRS, the percentage of pathogenic *ARID1B* mutations was as high as 76% of all identified SWI/SNF mutations (Wieczorek et al., 2013). Combining data from three studies (Tsurusaki et al., 2012; Santen et al., 2013; Wieczorek et al., 2013), NCBRS patients excluded, *ARID1B* is responsible for 62% of cases. In our previous study, we identified heterozygous truncating mutations in *ARID1B* in 16 patients with CSS and 3 patients with a diagnosis of NCBRS. The latter were clinically reevaluated and reclassified as having CSS in consequence of the molecular diagnosis. Interestingly, we observed ACC in 5/14 patients with *ARID1B* mutations (Wieczorek et al., 2013).

In the detailed clinical description of the patients from their initial study (Tsurusaki et al., 2012), Kosho et al. (2013) observed ACC and colpocephaly (disproportionate enlargement of the occipital horns of the lateral ventricles) in one out of four patients with mutations in *ARID1B*. The study of Santen et al. (2012) described colpocephaly in two patients. Thus this feature, if present, might be an additional diagnostic clue.

Santen et al. (2014) reviewed the *ARID1B*-related phenotype in detail, summarizing 60 patients from the literature and their own clinic. They defined the main clinical features of the *ARID1B* phenotype as ID, speech delay, a coarse facies, and hypertrichosis. Other relevant features were small fifth finger- or toenails (present in 81% of patients), short fifth finger (73%), feeding difficulties (65%), ACC (35%), seizures (23%), myopia (20%), and growth delay (19%). The authors state that the patients in this review mostly had a prior CSS diagnosis, leading to ascertainment bias for the most discernible CSS features such as brachydactyly of the fifth finger, nail hypoplasia, hypertrichosis, or sparse hair.

As mentioned before, the delineation of a common phenotype for *ARID1B* mutation carriers is hindered by ascertainment bias. All studies reported took a different approach to patient selection. Some were based on the detection of a *de novo* CNV, which led to the description of more patients with CNVs or mutations at the *ARID1B* locus or focused on a closely defined CSS cohort (Santen et al., 2012, 2013; Wieczorek et al., 2013), whereas other studies reported on cohorts defined by the common feature of autism spectrum disorder (ASD) or ID. The emerging picture is that the *ARID1B*-associated phenotype is highly variable with mild to severe ID, severe expressive speech impairment or absent speech (even in patients who may be capable of other forms of communication), corpus callosum anomalies in up to 1/3 of patients, autism in a significant fraction of patients, and a coarse facial appearance reminiscent of CSS in most patients. The cardinal CSS feature of hypoplastic fifth finger- or toenails was reported with varying proportions of up to 2/3 amongst patients with *ARID1B* mutations. Additional features, such as postnatal short stature, microcephaly, congenital heart defects, hearing impairment, visual impairment, hypertrichosis, sparse hair, and seizures were reported with varying frequencies.

*In conclusion*, we would like to modify the suggestion made by Santen et al. (2012) and propose that in addition to patients with an obvious CSS phenotype, a diagnosis of *ARID1B*-related SSRIDD should be considered in every patient with unexplained ID and speech impairment, especially if ACC and/or a coarse facial appearance are perceived (**Table 1**). This approach will certainly increase the diagnostic yield compared to a more broadly defined indication. As mentioned above, Hoyer et al. (2012), found mutations in *ARID1B* in 0.9% of their ID cohort. The frequency of *ARID1B* mutations in the UK 10K cohort of patients with unexplained ID was 0.4% (Grozeva et al., 2015). Wright et al. (2018) discovered 12 pathogenic mutations in *ARID1B* in a cohort of 1,133 patients with undefined developmental disorders, which corresponds to 1.06%. Keeping in mind that the use of targeted gene panels, as opposed to whole-exome (WES) or whole-genome sequencing, is the procedure of choice in most countries nowadays, the above clinical clues might aid in the choice of an accurate gene panel for patients with ID in the absence of a recognizable syndromic phenotype.

**Table 1:** Prominent clinical features that may distinguish the different SSRIDD phenotypes, sorted by causative genes.

Gene	Distinguishing features
*ARID1B*	Mild phenotypes without obvious CSS described
	Agenesis of corpus callosum in 1/3
	Trias: ID, absent speech, and ACC in non-syndromic cases
*ARID1A*	Nail hypo- or dysplasia of all toenails. Aplasia of toenails
	Facial asymmetry and scoliosis
	Severity of phenotype differs with grade of mosaic. May be very severe, comparable with *SMARCB1* and *SMARCE1* phenotypes
*ARID2*	Facial features reminiscent of NCBRS
	Possibly skeletal anomalies, especially wormian bones and plagiocephaly
*SMARCA2*	Typical NCBRS facial gestalt
	Swelling of interphalangeal joints
*SMARCA4*	Classic CSS with variable severity
*SMARCB1*	Large first toe with hypoplastic nail
	Dysplasia of thumbnails in one patient
	Hypoplasia may affect all toenails
	Prominent distal phalanges
	Large mouth with macroglossia
	Scoliosis
	Dextrocardia
*SMARCE1*	Hypo- or dysplasia of all toenails. Aplasia of toenails
	Long fingers with prominence of distal phalanx 5
	Scoliosis
*SOX11*	Absence of classic CSS facial features
	Shortened metacarpals beyond fifth ray
	Cutaneous syndactyly of toes 2/3, possibly also of fingers (one patient)
*DPF2*	Possibly craniosynostosis, especially sagittal


### Severe CSS Is Caused by *ARID1A* Germline Mutations or Mosaic Mutations Resulting in Milder Phenotypes

Truncating heterozygous mutations in *ARID1A* were first described in three subjects with a diagnosis of CSS by Tsurusaki et al. (2012). The phenotypes of these patients were described in detail in Kosho et al. (2013), who showed that mutations in *ARID1A* lead to a severe phenotype with congenital heart defects, feeding difficulties, bowel obstruction, and early severe respiratory complications warranting tracheostomy in two patients. All three patients had ACC, and one had additional brain malformations. One patient died from hepatoblastoma at age 2 years and another patient died at age 1 year from cardiac arrhythmia. The surviving patient has severe motor delay, absent speech, profound ID, and nail hypoplasia in addition to the above symptoms. He had sparse hair and some facial features reminiscent of CSS.

In our previous study (Wieczorek et al., 2013), we observed one patient with a nonsense mutation in *ARID1A*. As opposed to the patients described by Kosho et al. (2013), the phenotype of this patient consisted in mild ID, hypotonia, seizures, strabismus, hyperactivity, nail hypoplasia, hirsutism, abnormal corpus callosum, and a double ureter. His facial aspect was characterized by a low frontal hairline, thick and arched eyebrows, ptosis, low-set ears, and a thin upper and full lower vermillion border. He also had brachytelephalangy with small nails on all fingers and toes. This much milder phenotype compared to those observed by Kosho et al. (2013) may be explained by the fact that we had evidence of mosaicism for the *ARID1A* mutation in this patient. The analysis of DNA from blood lymphocytes does not allow the interpretation of the type of mosaicism, i.e., postzygotic or revertant mosaicism.

Mosaic mutations in *ARID1A* were also observed in four patients by Santen et al. (2013), who claimed somatic mosaicism as a possible mechanism in all published *ARID1A* patients and attributed the clinical variability to this observation. They saw a facial phenotype overlapping CSS but without sparse hair. Hypoplastic nails and brachydactyly were observed in four and three of their patients, respectively. Interestingly, they also observed delayed primary and permanent dentition in two and one of their patients, respectively. They observed mild to severe ID and moderate to severe speech delay in all of their *ARID1A* patients. Feeding problems and muscular hypotonia were seen in all four patients. One had seizures, one had myopia, and one had hearing impairment.

The phenotype as reviewed by Kosho et al. (2014) is variable with a tendency to the severe end of the spectrum, with mild (29%) to severe (57%) ID in all patients, CNS abnormalities in 88%, severe speech impairment in 83%, and cardiovascular (mostly septal defects), gastrointestinal (gastro-esophageal reflux, pyloric stenosis, anal atresia, Hirschsprung disease, or retrourethral fistula), and genitourinary complications (mainly cryptorchidism) in 57, 29, and 27%, respectively. Respiratory complications, such as laryngomalacia may occur and may lead to acute respiratory distress as mentioned above. Growth may be within normal limits, but severe postnatal growth retardation and microcephaly was documented for two individuals in Kosho et al. (2014). Finally, facial asymmetry is obvious in two patients with mutations in *ARID1A* depicted in Kosho et al. (2014) and may, if present, be an additional clue to diagnosis (**Table 1**).

### A CSS-Like Phenotype Is Caused by *ARID2* Mutations

Shang et al. (2015) first reported heterozygous, truncating mutations in *ARID2* in patients with ID. In addition to ID, they noted facial dysmorphisms (micrognathia or retrognathia, low-set or posteriorly rotated ears, epicanthal folds, down-slanting palpebral fissures, highly arched palate, and frontal bossing), behavioral problems, wormian bones, plagiocephaly, micro- and retrognathia, suggesting a syndromic condition, but they did not discuss an overlap with CSS. Bramswig et al. (2017) identified two heterozygous frameshift mutations in *ARID2* in two patients with a CSS-like condition. They reviewed the clinical data from Shang et al. (2015) and their own patients and found ID in all of them, muscular hypotonia in 4/5 and behavioral anomalies in 5/6. Facial features could be assessed for four patients and the authors noticed coarse facial features, frontal bossing or a large forehead, narrow palpebral fissures, a flat nasal bridge, and a prominent philtrum in all patients, as well as a broad nose with an upturned nasal tip and thick, anteverted alae nasi, and a large mouth in 3/4. A thick lower vermillion was seen in 4/4. All individuals described in Bramswig et al. (2017) also presented with very mild hypoplasia of the fifth fingernails and pronounced hypoplasia of the fifth toenails.

A patient reported by Van Paemel et al. (2017), who harbored an intragenic deletion spanning exons 3–5, presented with pre- and postnatal growth retardation, feeding problems, developmental delay, hearing impairment, hypermetropia, and facial features reminiscent of CSS, as well as sparse hair and hypoplasia of the fifth toenail. She also had skeletal features, namely bilateral hip dysplasia and club foot.

The authors of this review note a considerable overlap of the facial gestalt represented in the published photographs with NCBRS, namely a triangular face, thick anteverted alae nasi and a wide mouth with sharp corners of the mouth. A preliminary conclusion from these few reports is that mutations in *ARID2* cause a SSRIDD phenotype with facial features that overlap CSS as well as NCBRS, with hypoplastic fifth toenails and additional skeletal anomalies (**Table 1**).

### *SMARCA4* Mutations Cause Classic CSS

Twelve patients with mutations in *SMARCA4* have yet been described (Tsurusaki et al., 2012, 2014b; Santen et al., 2013). The clinical features have been summarized in Kosho et al. (2014): patients with *SMARCA4* mutations show a rather classic CSS phenotype with variable ID (mostly moderate to severe) and significant speech impairment that is somewhat milder than in the other patient groups (45% of patients learned to speak sentences, absent speech in only 36%). Structural CNS abnormalities (ACC, Dandy-Walker malformation and/or cerebellar hypoplasia) were reported in 86%, hypotonia in most patients, and cardiovascular (septal defects, persistent ductus arteriosus, valvular stenosis/atresia), gastrointestinal (gastro-esophageal reflux, pyloric stenosis, and frequently constipation), and genitourinary complications (cryptorchidism) in 42, 67, and 25%, respectively. Hearing impairment was present in 1/3 of patients and 45% had visual problems (mostly myopia). Feeding difficulties were reported in 11/12 patients, and five (of five reported) required tube feeding. Growth retardation was mostly mild, with height around -2 SD and head circumference around -2 SD to -3 SD. Most patients had typical facial features, but sparse scalp hair was observed only in 42%. Hypertrichosis, however, was present in all patients, indicating that this feature is more important for clinical diagnosis than sparse hair. Hypoplastic fifth fingers or toes and hypoplastic nails of the fifth ray were observed in 100% of these patients and 50% had hypoplasia of additional nails. 3/11 showed prominent interphalangeal joints, the NCBRS cardinal feature, and 5/10 had prominent distal phalanges. Scoliosis was only reported in one patient.

### *SMARCB1* Mutations Cause a Recognizable, Severe CSS-Like Phenotype

In our previous study (Wieczorek et al., 2013), we observed a *SMARCB1* mutation in one patient who showed a CSS facial phenotype with sparse hair, hypertelorism, downward slanting palpebral fissures, a short nose with anteverted nares, large mouth with macroglossia and low-set ears, nearly absent fifth fingernail, and hypoplasia of toenails III and V. She had moderate ID and seizures, and spoke no words at age 4 years. She also showed behavioral problems, such as hyperactivity and feeding problems. She was short and had microcephaly. She had a heart defect, auditory, and visual problems, as well as pyloric stenosis. She also had recurrent infections. In addition, we identified a mutation in *SMARCB1* in a patient initially diagnosed with NCBRS who was retrospectively reclassified as a CSS patient. The clinical details of this patient were described in Mari et al. (2015), and the clinical features are very similar to our initial patient, with growth retardation, microcephaly, ID, speech impairment (single words), and coarse facial features with thick eyebrows and a large mouth with macroglossia.

Kosho et al. (2013) described four individuals with mutations in *SMARCB1* in detail. The patients manifested a severe phenotype with profound ID, severely retarded psychomotor development (three patients were unable to sit unsupported and two patients could not walk by ages 2 and 7 years, respectively), seizures, absent speech and feeding problems. The patients whose pictures are shown also exhibited progressively coarse facial features and big first toes. One individual showed markedly rounded distal phalanges and nails in the sense of clubbing. Clubbed toes can also be appreciated each in one patient with a *SMARCA4* and an *ARID1A* mutation in Kosho et al. (2014). However, this finding is probably related to the presence of the respiratory complications bronchiectasis, bronchomalacia, and laryngomalacia in these patients (Kosho et al., 2014). Thus, clubbed toes and fingers caused by respiratory complications must be differentiated from prominent distal phalanges, which are a frequent skeletal trait in SSRIDD patients.

*SMARCB1* causes the most severe neurological complications within the SSRIDD spectrum with mostly severe ID, CNS abnormalities in 100% percent of patients (mainly ACC, but also cerebellar hypoplasia and Dandy-Walker malformation), seizures in 80% and absent speech in 80% of patients as reviewed by Kosho et al. (2014). Cardiovascular (septal defects, pulmonal artery stenosis, and/or dextrocardia), gastrointestinal (mainly gastro-esophageal reflux or pyloric stenosis), and genitourinary complications (cryptorchidism, horseshoe-kidney, or hydronephrosis) were reported in 40, 70, and 45%, respectively. Dextrocardia was observed in two individuals (Santen et al., 2013; Kosho et al., 2014). Feeding difficulties are reported for all patients, requiring long-term tube feeding in most. Most patients show postnatal growth retardation of about -3 to -5 SD for height and -2.5 to -4 SD for OFC. Scoliosis is also a prominent feature, reported in 78% and in many of them severe (Kosho et al., 2014).

Notably, the progressive and marked coarseness of the face, as well as the big first toe are features that are also observed in NCBRS. The overlap with NCBRS is supported by the description of a patient with a *SMARCB1* mutation who also developed schwannomatosis due to additional somatic loss of both *NF2* copies (Gossai et al., 2015). This adult patient shows marked coarseness of the face reminiscent of NCBRS, a large first toe with nail hypoplasia as well as deformities of small joints on the hands and feet. The hallmark feature of NCBRS, “prominent interphalangeal joints”, was reported in 44% of patients with *SMARCB1* mutations by Kosho et al. (2014).

*To summarize*, non-truncating mutations in *SMARCB1* cause a severe CSS-like phenotype with a recognizable facial gestalt that includes hypertelorism, thick eyebrows, a depressed and broad nasal bridge and anteverted nares, and a large mouth with macroglossia and progressive coarseness of the face, as well as sparse scalp hair and hypertrichosis. Patients show hypoplasia and dysplasia of nails, mostly concerning more than the fifth rays and a large and broad first toe. Severe scoliosis is also frequently observed in patients with *SMARCB1* mutations (**Table 1**). As the phenotype progresses, there is a marked overlap with NCBRS, which places the *SMARCB1*-related phenotype in between the known CSS and NCBRS phenotypes and again challenges the established notion that NCBRS and CSS are distinct entities.

### The Phenotype Caused by *SMARCE1* Mutations Is Similar to *SMARCB1*-Associated SSRIDD

*SMARCE1* mutations have been described in six patients so far (Tsurusaki et al., 2012; Santen et al., 2013; Wieczorek et al., 2013; Zarate et al., 2016). The phenotype is similar to *SMARCB1*-associated SSRIDD and located at the severe end of the SSRIDD spectrum. ID is moderate to severe and patients also present with seizures, CNS abnormalities (mainly ACC, but also cerebellar hypoplasia and Dandy-Walker malformation) and severe speech impairment Kosho et al. (2014). There may be pre- and postnatal growth retardation of up to -5.6 SD for height and up to -4.6 SD for OFC (Kosho et al., 2014). The facial aspect is that of severe CSS with sparse hair, coarse facial features, and full lips. The fingers may appear long and slender, with a prominent distal phalanx 5 as depicted in Kosho et al. (2014). Notably, the toenails of patients with *SMARCE1* mutations may not only be hypoplastic but also dysplastic, as indicated by a thick and discolored aspect. Moreover, the hypo- or even aplasia of toenails may be most marked on the first toe instead of the fifth, as depicted in Kosho et al. (2013), Wieczorek et al. (2013), and Zarate et al. (2016) (**Table 1**). One patient in Zarate et al. (2016) presented with absence of the second fingernail of the right hand. The facial features of patients 2 and 3 in Zarate et al. (2016) again overlap with features of NCBRS.

### Classic NCBRS Is Caused by Mutations in *SMARCA2*

Heterozygous mutations in the *SMARCA2* gene were described as the cause of NCBRS (Van Houdt et al., 2012). NCBRS is without doubt the most recognizable and the most homogeneous phenotype within the SSRIDD spectrum, clinically as well as molecularly. So far, mutations in *SMARCA2* have only been observed in patients with a clear NCBRS phenotype, and patients who had been clinically diagnosed with CSS and found to harbor a *SMARCA2* mutation had to be reclassified as having NCBRS upon second look (Wieczorek et al., 2013; Tsurusaki et al., 2014b).

Hallmark features of the disorder are ID with marked expressive speech delay, sparse hair, short stature, microcephaly, a recognizable facial gestalt, brachydactyly, prominent interphalangeal joints, and seizures (Nicolaides and Baraitser, 1993; Sousa et al., 2009). The face is triangular and becomes coarse with time, and most patients have a large open mouth with a full lower lip and sometimes a protruding tongue. The broad mouth leads to a wide, impish smile. The nose is rather long with a narrow bridge and the nares are anteverted. It also appears to the authors of this paper that the columella is low hanging in many patients, leading to a pear shaped aspect of the nose especially in older subjects. The eyes tend to slant downward in some patients. The eyebrows are often bushy and curved. There may be wrinkling of the skin and sagging of the periorbital skin. The phenotype may be harder to discern at very early ages, and other authors already noted a fair overlap with Williams syndrome (Sousa et al., 2009, 2014).

Sousa et al. (2014) reviewed the clinical characteristics of 61 patients with molecularly proven NCBRS and, interestingly, 10 of these patients were aged 17 years or older and were followed up over several years, allowing for a very comprehensive description of the phenotype and its evolution through childhood. They noted that 33% of patients were born small for gestational age, and 23% had congenital microcephaly. Postnatal short stature was observed in 54% and failure to thrive in 52%. Postnatal microcephaly was observed in 65%. ID was mild in 18%, moderate in 36%, and severe in 54%. Speech delay was common, with speech decline in 21% and absent speech in 32%. Seizures occurred in 64% of patients. Sparse scalp hair was the most prevalent feature seen in 97% of patients. Facial features were noted to be coarse in 77% and the coarseness was progressive in 58%. Progression was seen in follow up patients also for sparseness of hair and for the joint swellings, which can lead to restricted mobility. The CSS hallmark feature of small or absent distal phalanges and/or fingernails of the fifth fingers is not a feature of NCBRS. Although there is significant overlap with CSS, the facial gestalt of NCBRS, the prominent interphalangeal joints, and the absence of nail hypoplasia may serve to distinguish the entities clinically (**Table 1**). However, prominent interphalangeal joints have also been documented in 1/3 of CSS patients (Kosho et al., 2014) and nail hypoplasia can be very subtle in CSS, making the clinical diagnosis challenging in patients with a less classic presentation.

### A Novel SSRIDD Caused by Mutations in *SOX11*

*De novo* mutations in *SOX11* in patients with a CSS-like phenotype were first described in Tsurusaki et al. (2014a). Patients with mutations in or deletions of *SOX11* showed clinical features overlapping with CSS such as developmental delay, ID, microcephaly, feeding problems, and hypoplastic fifth fingers and finger-/toenails. Ocular anomalies were frequently reported in *SOX11* patients, such as microphthalmia, hypermetropia, strabismus, and oculomotor apraxia. The patient described by Okamoto et al. (2017) had a heart defect and the patient described by Khan et al. (2018) had cleft palate. Many of the reported patients showed cutaneous syndactyly of the toes 2–3. One patient (case 4 in Hempel et al., 2016) shows a severe shortening of the metacarpals, especially of the fifth ray, and cutaneous syndactyly of the fourth and fifth fingers, reaching up to the proximal interphalangeal joint of the fifth finger (observed on printed photographs). Thus, the finding of syndactyly might set patients with *SOX11* mutations apart from patients with other forms of SSRIDD (**Table 1**). Interestingly, the patient described by Khan et al. (2018) shows prominent interphalangeal joints, reminiscent of NCBRS. Hempel et al. (2016) noted that although the patients in their study had features compatible with CSS, none of their patients had a clinical diagnosis of CSS prior to the molecular analysis performed, and they attributed this fact to the absence of typical CSS facial features in their patients. They also pointed out that the symptoms in their patients overlapped with other syndromes, such as mosaic trisomy 9 or deafness-onychodystrophy–osteodystrophy–mental retardation syndrome.

*In conclusion*, mutations in *SOX11* cause a phenotype that fits into the SSRIDD spectrum, but can hardly be defined as CSS. Since SOX11 is a downstream target of the SWI/SNF complex, instead of a complex component, it is not surprising that the phenotype differs more strongly from CSS than other forms of SSRIDD caused by mutations in SWI/SNF complex genes.

### A Novel SSRIDD Caused by Mutations in *DPF2*

The phenotype of patients with heterozygous mutations in *DPF2* fits very well into the SSRIDD spectrum. Vasileiou et al. (2018) described eight patients with mutations in this gene and all of them showed global developmental delay, moderate to borderline ID, and speech delay. Interestingly, hypoplasia of the fifth toenails was observed in all individuals, and some showed hypoplasia of further toenails and/or fingernails. Generalized or fifth-finger brachydactyly was seen in four children and fifth-finger clinodactyly in three. As in CSS, most individuals had coarse facial features. Sparse scalp hair, down-slanting palpebral fissures, thick or small alae nasi, a short or broad philtrum, and large, prominent, low-set and/or posteriorly rotated ears were seen in six patients each. Other symptoms (in at least three patients) were a prominent forehead, a broad nose, a wide mouth, a thin upper and thick lower lip, thick eyebrows, broad thumbs, and prominent fetal fingertip pads. Five of eight children presented with short stature, four individuals showed muscular hypotonia, four had hearing impairment, and five had feeding problems. Behavioral anomalies varied across individuals and autism was suspected in one. No corpus callosum anomalies were noted upon cerebral MRI in three children, however, atrophy in the right cerebellar hemisphere, a small pituitary gland, and Arnold-Chiari malformation I were seen in one patient each. Interestingly, three of eight children presented with sagittal craniosynostosis or trigonocephaly (**Table 1**).

### Differential Diagnoses to SSRIDD

Bramswig et al. (2015) studied 10 unsolved CSS cases by WES and found interesting differential diagnoses in five of them, including Wiedemann-Steiner syndrome, Kabuki syndrome, and Adams-Oliver syndrome. They also found one possibly pathogenic heterozygous *de novo* mutation in *GRIN2A* causing a dominant epilepsy-aphasia spectrum disorder (MIM: 245570), and one in *SHANK3* which is involved in the pathogenesis of Phelan-McDermid syndrome (MIM: 606232), which can be associated with dysplastic toenails and other less specific features overlapping CSS (i.e., ID, feeding problems, speech delay).

Mutations in the X-chromosomal *PHF6* gene, which normally cause Borjeson-Forssman-Lehmann syndrome, were identified in two girls with a phenotype partially overlapping CSS (Wieczorek et al., 2013).

Campeau et al. (2014) noted considerable phenotypic overlap between patients with *SMARCB1* mutations and DOORS syndrome (Deafness, Onychodystrophy, Osteodystrophy, mental Retardation, Seizures) (OMIM 220500). They stated that all major characteristics of DOORS syndrome, except epilepsy, were present in two individuals with a *de novo SMARCB1* mutation previously described in CSS. However, the patients with the *SMARCB1* mutation additionally showed CNS malformations, respiratory complications, and scoliosis, as well as limb and facial features that differed from the molecularly proven DOORS syndrome patients in their study. Most importantly, they did not have 2-oxoglutaric aciduria.

Mutations in the SWI/SNF-complex-related gene *ADNP* (Activity-Dependent Neuroprotector Homeobox; OMIM 611386) cause a syndromic form of autism called Helsmoortel-van der Aa syndrome (OMIM 615873). So far, 11 patients with this condition have been described (Helsmoortel et al., 2014; Vandeweyer et al., 2014), and although these patients do not show a coarse face, sparse hair or hypertrichosis, they present several clinical features overlapping SSRIDD. All patients had autism, developmental delay, ID and speech impairment, including one patient with absent speech. Almost all children have hand abnormalities, including clinodactyly, polydactyly, fetal finger pads, and, most importantly, small fifth fingers, and/or prominent interphalangeal joints, and distal phalanges. Seven out of 11 patients had feeding problems, and six have gastro-esophageal reflux. More than half of the patients have visual problems, such as hypermetropia or strabismus. Facial features include ptosis, abnormal slant of palpebral fissures, wide nasal bridge, upturned nasal tip, and a thin upper lip, and ear abnormalities, such as small low-set ears, protruding cup-shaped ears, and bilateral helical indentation. Five out of eight children had CNS abnormalities seen on MRI, but none had ACC (Vandeweyer et al., 2014). The photographs published by Helsmoortel et al. (2014) show that although some facial features overlap with the SSRIDD spectrum, the phenotype is distinct from CSS. More clinical reports of patients with mutations in *ADNP* are needed to determine whether Helsmoortel-van der Aa syndrome should be regarded as part of the SSRIDD spectrum due to the clinical overlap and the functional interaction between ADNP and the SWI/SNF complex (Vandeweyer et al., 2014). At this point, however, we would classify Helsmoortel-van der Aa syndrome as an important differential diagnosis to SSRIDD.

### SWI/SNF Genes in Autism Spectrum Disorder

Autism has been described in patients with SSRIDD, especially among patients with mutations in *ARID1B*. This observation is mirrored by the findings of De Rubeis et al. (2014), who found *ARID1B* among the high risk genes for autism, defined by two or more *de novo* loss-of-function mutations among 2270 children with ASD. The finding was replicated by Iossifov et al. (2014), who found three *de novo* variants in *ARID1B* among 2509 patients with autism and none in unaffected siblings. Recent studies compared the probability of recurrent *de novo* mutation in individual genes among autism probands, and found *ARID1B* to be the second most likely gene to carry a *de novo* mutation in a patient with autism (Stessmann et al., 2017; Yuen et al., 2017). It is arguable that the patients in these studies may have a SSRIDD phenotype, however, autism seems to have been a prominent feature in these patients to be selected for the cohorts. The association between autism and SSRIDD is supported by a recently published Arid1b mouse model, which demonstrated autism-like behaviors in Arid1b-haploinsufficient mice (Shibutani et al., 2017).

Interestingly, although mutations in other SWI/SNF genes were observed in the studies of De Rubeis et al. (2014) and Iossifov et al. (2014), no other SSRIDD gene was identified as a high-risk ASD gene. This either indicates that mutations in *ARID1B* may contribute to a higher risk for autism than mutations in the other SSRIDD-associated SWI/SNF genes, or it may simply reflect the high frequency of occurrence of *ARID1B* mutations among patients with developmental disorders (Deciphering Developmental Disorders Study [DDD], 2017; Wright et al., 2018).

*In conclusion*, large genetic studies in autism cohorts (De Rubeis et al., 2014; Iossifov et al., 2014; Stessmann et al., 2017; Yuen et al., 2017) have identified *ARID1B* and other genes involved in chromatin remodeling to be frequently mutated in children with ASD. However, most patients with such mutations likely suffer from syndromic disorders, which manifest with ASD, amongst other complaints. Thus, instead of identifying SWI/SNF genes such as *ARID1B* and *ADNP* purely as autism risk genes, the above studies underlined the high prevalence of ASD in children with SSRIDD and related disorders, which is an important observation. Hence, a child diagnosed with ASD, in whom a mutation in a SWI/SNF gene is identified, should receive a directed clinical evaluation regarding the other manifestations of SSRIDD, while every child diagnosed SSRIDD should receive formal testing for autistic traits in order to improve clinical management.

## Mutational Landscapes of Genes Involved in SSRIDD

### The *ARID* Gene Family

**FIGURE 3:**
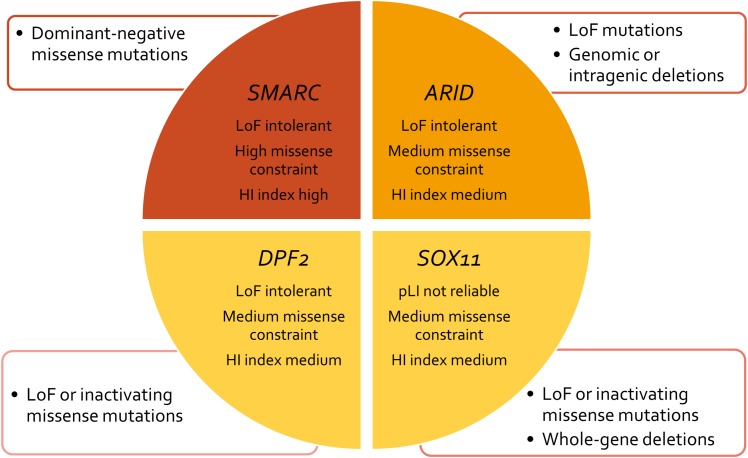
Concept chart of SSRIDD genes and their mutational characteristics and constraint metrics. LoF, loss of function; HI, haploinsufficiency; pLI, probability of a gene being LoF intolerant.

The term “ARID” stands for “AT-rich domain containing protein” and basically denotes a group of proteins that share the ARID effector domain. The *ARID* genes involved in SSRIDD are *ARID1B* (OMIM 614556), *ARID1A* (OMIM 603024), and *ARID2* (OMIM 609539). Mutations in these *ARID* family genes are heterozygous truncating, loss-of-function mutations, or intragenic/whole-gene deletions, with the exception of very few *de novo* missense mutations in *ARID1B*. Whenever parental samples were available, pathogenic mutations in these genes were shown to have occurred *de novo*. Probable gonadal mosaicism for an ARID1B mutations has been described in one family with three affected siblings (Ben-Salem et al., 2016). Based on the fact that mutations in the *ARID* gene family are almost entirely truncating, the pathomechanism of mutations in this gene family is most likely haploinsufficiency (**Figure 3**).

**FIGURE 4:**
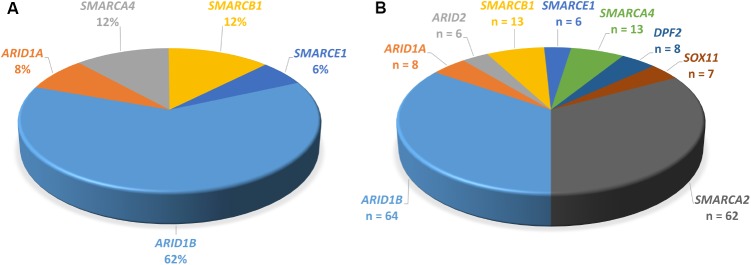
Proportions of genes mutated in SSRIDD. **(A)** *ARID1B* is the most commonly mutated gene in CSS. Pie-chart of percentages of genes mutated in patients with CSS from studies Tsurusaki et al. (2012), Santen et al. (2013), Wieczorek et al. (2013), and Tsurusaki et al. (2014b) (*n* = 103). **(B)** Pie-chart of all point mutations in the nine genes associated with SSRIDD described in the literature. Number of patients with a mutation in each gene. Numbers serve as an overview and do not reflect mutation detection rates, because the underlying studies used very differentially selected patient collectives (*n* = 190).

**FIGURE 5:**
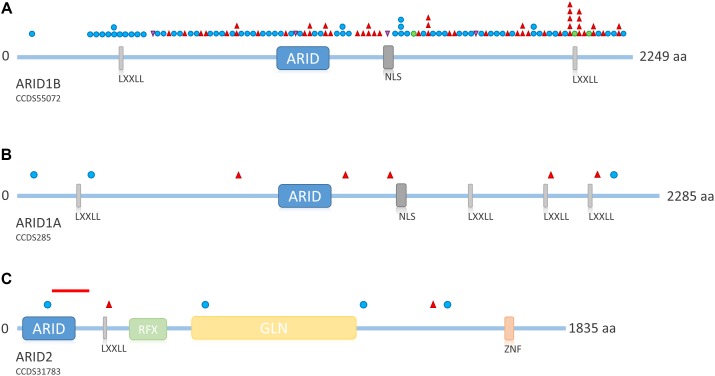
Overview of ARID family protein structure and distribution of mutations across the proteins. **(A)** Schematic representation of ARID1B. Mutations depicted from LOVD *ARID1B* database. Protein structure according to uniprot ID Q8NFD5. **(B)** Schematic representation of ARID1A. Mutations according to Tsurusaki et al. (2012); Santen et al. (2013), and Wieczorek et al. (2013). Protein structure according to uniprot ID O14497. **(C)** Schematic representation of ARID2. Mutations according to Shang et al. (2015) and Bramswig et al. (2017). The red line indicates the intragenic deletion reported by Van Paemel et al. (2017). Protein structure according to uniprot ID Q68CP9. Blue circle, frameshift mutation; red triangle, stop mutation; green circle, missense mutation; purple triangle, splice site mutation. LXXLL, nuclear receptor recognition motif; NLS, nuclear localization signal; RFX, RFX-type winged-helix; GLN, glycine rich region; ZNF, zinc finger.

Mutations in *ARID1B* are by far the most common cause of SSRIDD, and 64 patients with syndromic ID or CSS and mutations in *ARID1B* have yet been described in the literature (Sim et al., 2015; **Figure 4**). Additionally, 25 patients with deletions of the *ARID1B* locus have been published (reviewed in Santen et al. (2014). The LOVD *ARID1B* database contains 98 different pathogenic or likely pathogenic mutations in 119 patients (https://databases.lovd.nl/shared/variants/ARID1B; accessed March 8, 2018, 9:00 a.m.) (Supplementary Table S2). These mutations consist of 57 frameshift (58%), 34 stop (35%), 3 missense (3%), and 4 splice site (4%) mutations (Supplementary Figure S1). Causative mutations in *ARID1B* are distributed across the entire protein and not restricted to a specific domain (**Figure 5A**). Most documented mutations are private, but some recurrent mutations (occurrence documented in more than one patient) have been observed, especially c.5968C>T, p.(Arg1990^∗^) (five patients) and c.6038G>A, p.(Trp2013^∗^) (four patients) (**Figure 5A**). Most missense variants and in-frame indels in *ARID1B* that are annotated in the LOVD database are estimated not to affect protein function (*n* = 13). From our own experience, non-truncating variants are mostly unlikely to affect protein function, especially variants within exon 1, which is prone to missense variants, indels and artifacts due to its high GC content and repetitive sequence. However, two *de novo* missense mutations in *ARID1B* have been described in patients with non-syndromic short stature (Yu et al., 2015), and these were estimated to be likely pathogenic by the authors. A third missense variant is annotated in LOVD as a likely pathogenic *de novo* mutation (Supplementary Table S2 and **Figure 5A**). These likely pathogenic variants were located in the C-terminal half of the gene. Thus, segregation analysis should be performed for every detected missense variant that cannot be ruled out through database research.

*ARID1A* mutations are a much rarer cause of SSRIDD and only eight heterozygous truncating mutations (three frameshift and five stop mutations; **Figure 5B**) have been described as yet (Tsurusaki et al., 2012; Santen et al., 2013; Wieczorek et al., 2013). Mutations are spread over the entire gene/protein without any hotspots (**Figure 5B**). Mutations were identified as mosaic by ourselves (Wieczorek et al., 2013) and Santen et al. (2013), who found all of their four mutations in *ARID1A* to be mosaic. The latter authors claimed that possibly all mutations in *ARID1A* are mosaic, since according to Gao et al. (2008), heterozygous truncating variants in *Arid1a* are embryonically lethal in mice. Kosho et al. (2014) retrospectively claimed the mutations in *ARID1A* from their initial study (Tsurusaki et al., 2012) to be likely mosaic as well, as suggested by Santen et al. (2013) on the basis of the depicted electropherograms. We conclude that, most likely, truncating mutations in *ARID1A* are mosaic, and that the degree of mosaicism in different tissues underlies the variable phenotypic expression of *ARID1A*-associated SSRIDD.

Recently, heterozygous truncating mutations in *ARID2* have been described in two patients with a CSS-like phenotype (Bramswig et al., 2017), complementing the description of *ARID2* as a gene for ID by Shang et al. (2015). The findings were confirmed by the report of a deletion of 105 kb at 12q12, spanning exons 3–5 of *ARID2*, in a patient with CSS (Van Paemel et al., 2017). Overall, four frameshift and two missense mutations and one intragenic deletion have been observed (**Figure 5C**).

### Theoretical Reflections on the Predominance of *ARID1B*

The predominance of *ARID1B* mutations across several CSS cohorts (Tsurusaki et al., 2012, 2014b; Santen et al., 2013; Wieczorek et al., 2013), and also in unselected ID cohorts (Hoyer et al., 2012; Grozeva et al., 2015; Wright et al., 2018), raises the question why this gene is so often affected by mutations. Four hypotheses apply: (i) There might be a higher mutation rate of this gene compared to other developmentally important genes; (ii) mutations in *ARID1B* might be associated with a relatively higher survival rate than mutations in other developmental genes; (iii) mutations in *ARID1B* might confer a positive selection bias in sperm; (iv) the mutational mechanism is less restrictive compared to the *SMARC* gene family.

Lek et al. (2016) analyzed the exomes of 60,706 humans and identified genes which underlie mutational constraint. These genes are, in general, important developmental genes which could be shown to harbor fewer mutations than would be statistically expected. The deviation from expectation was quantified with a *Z* score and for protein-truncating variants with an “expectation-maximization algorithm”, which quantifies the probability of a gene being loss of function intolerant (pLI). A loss of function intolerant gene is expected to have a pLI of ≥0.9. This was the case for 3,230 genes of 18,225 genes analyzed. The *ARID* family genes all exhibit a pLI of 1.0, indicating loss of function intolerance (**Table 2**). The average *Z* score for missense variants of this gene family is 3.12, indicating medium missense constraint (**Table 2**). Another metric of haploinsufficiency is the haploinsufficiency score (HI index) developed by the group of Huang et al. (2010). HI indices of <10% indicate a gene is more likely to exhibit haploinsufficiency, while ranks >90% indicate a gene is very unlikely to exhibit haploinsufficiency. HI indices between 10 and 90% are less unequivocal. According to the authors, genes with an HI index in the lowest quartile (<25%) are most likely to exhibit haploinsufficiency. HI indices can be accessed at https://decipher.sanger.ac.uk/. The *ARID* family genes exhibit HI scores between 9.63 (*ARID1A*) and 14.17 (*ARID1B*), with an average of 11.60 (**Table 2**). These moderate HI scores together with the medium missense constraint might reflect the fact that *ARID1B* is relatively tolerant against missense mutations, which is why we observe, almost exclusively, truncating variants as causative mutations (**Figure 3** and Supplementary Figure S1). These constraint metrics argue against higher mutability as the reason for the high proportion of *ARID1B* mutations in the SSRIDD cohorts and rather support hypothesis number two, a weaker negative selection effect of mutations in *ARID1B* compared to other developmental genes.

**Table 2:** Haploinsufficiency scores and constraint metrics of SSRIDD genes^∗^.

Gene	HI%	pLI	*Z* score missense
*ARID1A*	9.63	1.00	4.10
*ARID1B*	14.17	1.00	3.39
*ARID2*	11.01	1.00	1.87
**Group average**	**11.60**	**1.00**	**3.12**
*SMARCA2*	1.60	1.00	5.57
*SMARCA4*	2.94	1.00	8.64
*SMARCB1*	8.06	1.00	4.51
*SMARCE1*	11.97	1.00	2.96
**Group average**	**6.14**	**1.00**	**5.42**
*SOX11*	20.02	0.34	4.25
*DPF2*	10.25	1.00	3.30


*^∗^Lek et al. (2016) (Exome Aggregation Consortium). Bold values represent average values for the ARID/SMARC gene families.*

Paternal age effect due to selfish spermatogonial selection is a known and well established concept, leading to an increased mutation rate in genes such as *FGFR3* or genes involved in the MAPK pathway in the sperm of older men (aged >40) (Goriely and Wilkie, 2012; Kong et al., 2012; Maher et al., 2014). We combined data on paternal age from the studies of Mari et al. (2015) and our own (Wieczorek et al., 2013) and we observed a medium paternal age of 36 years among the fathers of 21 CSS patients (with mutations in *ARID1B, ARID1A, SMARCA2, SMARCB1*, and *SMARCE1*). Seven of these 21 fathers were aged over 40 years (27%). Reliable data on the average paternal age in the normal population are rare. Data from one review article suggest average paternal ages between 32 and 34 years in different European populations (Willführ and Klüsener, 2018). Although this is an interesting observation, it is not unsuspected, given the higher incidence of *de novo* mutations in the offspring of older parents, fathers especially (Yuen et al., 2016) and the numbers presented here are far too small to draw any definite conclusions. Since this observation is not restricted to *ARID1B* but concerns all SWI/SNF genes, paternal age does not explain the predominance of *ARID1B* mutations. Additionally, the loss-of-function mutations which are observed in the *ARID* family genes would be expected to underlie negative selection (Lek et al., 2016), arguing against selfish spermatogonial selection.

Finally, the loss-of-function mutations in the *ARID* family genes are distributed across the entire gene and lead to haploinsufficiency, while very specific mutations in the *SMARC* genes are thought to confer a dominant negative effect and are restricted to segments of the gene that encode important protein domains. Thus, causative mutations in the *ARID* genes might be more likely to occur than the specific mutations in the *SMARC* genes. However, this comparison only holds true within the SSRIDD spectrum and does not explain the predominance of *ARID1B* mutations over other developmental genes such as *ADNP* or *KAT6A*, also frequently affected by loss-of-function mutations.

### The *SMARC* Gene Family

The term “SMARC” stands for “swi/snf-related, matrix-associated, actin-dependent regulator of chromatin” and designates a protein family that shares homology with the yeast swi/snf proteins. The *SMARC* genes that are involved in SSRIDD are *SMARCA2* (OMIM 600014), *SMARCA4* (OMIM 603254), *SMARCB1* (OMIM 601607), and *SMARCE1* (OMIM 603111). Virtually all mutations in these genes occur *de novo*, however, autosomal dominant inheritance was documented for one *SMARCE1* mutation (Santen et al., 2013). The *SMARC* family genes all exhibit a pLI of 1.0, indicating loss-of-function intolerance (**Table 2** and **Figure 3**). The average *Z* score for missense variants of this gene family is 5.42, indicating high missense constraint (**Table 2**). The *SMARC* family genes exhibit HI scores of 1.6 (*SMARCA2*) to 11.97 (*SMARCE1*), with an average of 6.14, indicating haploinsufficiency (**Table 2**).

Mutations in the *SMARC* gene family that cause SSRIDD are almost all non-truncating, i.e., missense mutations or in-frame deletions. Since almost no patients with truncating germline mutations in the *SMARC* genes are known and because of the evolutionary conservation of these genes, which is reflected in the above statistical metrics, one might conclude that germline truncating mutations in these important developmental genes would be embryonic lethal. However, the results of recent research refute this conclusion.

**FIGURE 6:**
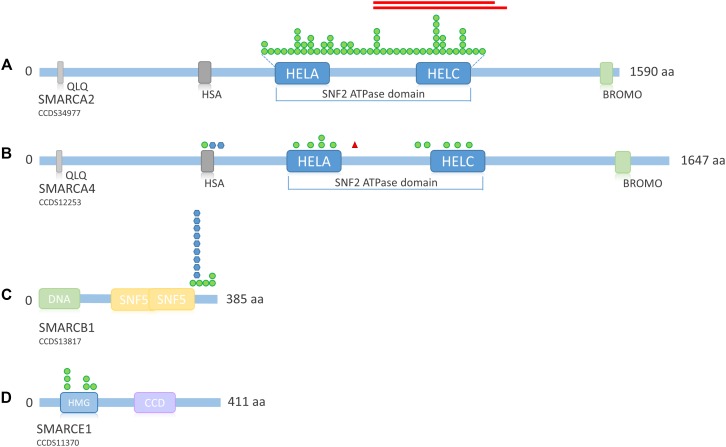
Overview of *SMARC* family protein structure and distribution of mutations across the proteins. **(A)** Schematic representation of SMARCA2. Mutations according to Sousa et al. (2014), Sánchez and Rojas (2017). Protein structure according to uniprot ID P51531. **(B)** Schematic representation of SMARCA4. Mutations according to Tsurusaki et al. (2012), Santen et al. (2013), Tsurusaki et al. (2014b), and Tzeng et al. (2014). Protein structure according to uniprot ID P51532. **(C)** Schematic representation of SMARCB1. Mutations according to Tsurusaki et al. (2012), Santen et al. (2013), Tsurusaki et al. (2014b), Wieczorek et al. (2013), and Gossai et al. (2015). Protein structure according to uniprot ID Q12824. **(D)** Schematic representation of SMARCE1. Mutations according to Tsurusaki et al. (2012), Santen et al. (2013), Wieczorek et al. (2013), and Zarate et al. (2016). Protein structure according to uniprot ID Q969G3. Green circle, missense mutation; blue hexagon, in-frame deletion; red triangle, stop mutation; red bars, in-frame deletion. QLQ, Gln, Leu, Gln motif; has, RFX-type winged-helix; HELA, ATP-Helicase; HELC, Helicase C-terminal; Bromo, bromodomain; HMG, high mobility group box; CCD, coiled coil domain.

Mutations in *SMARCA2* causing NCBRS are heterozygous missense or in-frame deletions located within the SNF2 ATPase domain (encoded by exons 15–25; Sousa et al. (2014) (**Figure 6A**). So far, 50 different missense mutations and two in-frame deletions have been identified in 62 patients (Tsurusaki et al., 2012; Van Houdt et al., 2012; Wolff et al., 2012; Santen et al., 2013; Wieczorek et al., 2013; Sousa et al., 2014; Sánchez and Rojas, 2017). Wolff et al. (2012) and Tsurusaki et al. (2012) also identified in-frame intragenic deletions of the *SMARCA2* gene (exons 20–26 and exons 20–27, respectively).

According to Van Houdt et al. (2012) the facts that deletions encompassing the entire *SMARCA2* gene do not cause NCBRS, that mice lacking functional *Smarca2* do not present with major developmental abnormalities, and that the solely non-truncating mutations in NCBRS patients are located exclusively in the SNF2 ATPase domain suggest that these mutations most likely do not lead to haploinsufficiency, but rather have a specific dominant-negative or gain-of-function effect (Van Houdt et al., 2012 and references therein). Sousa et al. (2014) pointed out that according to functional studies in yeast, missense mutations in the ATPase domain most likely lead to a structurally normal, but inactive BAF complex with normal localization at the chromatin, which might exert a dominant-negative effect on the complex. Our own search in the DECIPHER database (https://decipher.sanger.ac.uk/, accessed March 12, 2018) revealed that a whole-gene deletion and one deletion encompassing the N-terminus of the *SMARCA2* gene were inherited from normal parents and thus not associated with any obvious phenotype. The whole-gene deletion was inherited from the mother and the N-terminal deletion was inherited from the father, ruling out imprinting. This finding further supports the idea that *SMARCA2* is not haploinsufficient, although its HI rank would suggest so (**Table 2** and **Figure 3**).

Heterozygous *SMARCA4* mutations cause 12% of CSS cases (**Figure 4A**) and have been described in 13 patients (**Figure 4B**). The heterozygous missense mutations are located within or in close proximity to the HSA domain or the SNF2 ATPase domain (**Figure 6B**). A study by Hodges et al. (2018) showed that heterozygous missense mutations in the ATPase domain of *SMARCA4* have a dominant-negative effect. They analyzed the effect of missense mutations observed in cancer cells, including the mutation c.2654G>A, p.(Arg885His), which has also been described in a patient with CSS (Tsurusaki et al., 2014b). Hodges et al. (2018) observed reduced DNA accessibility at a multitude of enhancer sites as a result of the studied mutations. This effect could not be observed after generation of a conditional heterozygous knock-out allele in mouse, although reduction of Smarca4 protein levels by 50% was shown. Therefore, the authors concluded that the loss of chromatin accessibility is the result of a dominant-negative effect.

Germline truncating *SMARCA4* mutations cause a very rare tumor predisposition syndrome called Rhabdoid Tumor Predisposition Syndrome 2 (RTPS2; OMIM 613325), which is characterized by the early occurrence of small-cell carcinomas of the ovary hypercalcemic type (SCCOHT) or rhabdoid brain tumors. Interestingly, one patient with concomitant SCCOHT at age 13 years and mild CSS has recently been described (Errichiello et al., 2017). The phenotype was caused by a germline heterozygous nonsense mutation within the SNF2 ATPase region of *SMARCA4*, downstream of the ATP-binding helicase domain [c.2935C>T, p.(Arg979^∗^)], in combination with a somatic frameshift mutation as a second hit in the ovary (Errichiello et al., 2017). This finding indicates that truncating mutations located downstream of the ATP-binding helicase domain (amino acids 766–931), might also confer a dominant-negative effect. However, the authors showed that the nonsense mutation was issue to partial nonsense mediated decay, which might also explain the mild CSS phenotype in their patient.

This hypothesis is also in line with the published C-terminal deletions found in *SMARCA2*, which delete the C-terminal helicase domain, but not the ATP-binding helicase domain (Tsurusaki et al., 2012; Wolff et al., 2012) (**Figure 6A**). Thus, a truncated SMARCA2 or SMARCA4 protein that possesses the ATP-binding helicase domain, but lacks a functional C-terminal helicase domain might also elicit a dominant-negative effect, however, this theory remains to be proven.

There are considerable structural differences between the SMARC subfamilies, while genes within the same subfamily are highly homologous (**Figure 6**). In this context, it is interesting to note that the two genes from subfamily A (*SMARCA2* and *SMARCA4*), which share high homology, cause such distinct phenotypes within the disease spectrum (NCBRS vs. CSS). This is owed to the fact that SMARCA2 and SMARCA4 are the two mutually exclusive ATPase subunits of the SWI/SNF complex. Keeping in mind that the differentially composed complexes have overlapping but also specific functions in mammalian development (Kadoch and Crabtree, 2015), the differential phenotypic outcome of mutations in these highly homologous subunits becomes comprehensible. Possibly, their homology is also the key to the pathomechanism of mutations in these genes. Since either SMARCA2 or SMARCA4 are present as a catalytic subunit in SWI/SNF complexes, it is conceivable that a loss of one copy of either gene might in part be compensated by increased use of the other homolog, thus preventing haploinsufficiency, whereas a dominant-negative mutation, leading to a structurally normal but functionless complex, would probably disable this compensatory effect. Although it has been previously shown that Smarca4 (Brg1) was essential for early embryonic development in mice and could compensate for the nonessential Smarca2 (Brm) (LeGouy et al., 1998), but not the other way around, a study by Strobeck et al. (2002), showed that SMARCA2 (BRM) could in fact compensate for loss of SMARCA4 (BRG-1) in a retinoblastoma cell line. Wilson et al. (2014) also found that the compensatory formation of a SMARCA2 containing complex is associated with oncogenic drive in *SMARCA4* mutated cancer cell lines. Thus, the compensatory properties of either subunit seem to depend on the developmental time-point.

The pathomechanism of mutations in *SMARCB1* and *SMARCE1* is unknown. Since all described mutations in SSRIDD patients were also missense mutations or in-frame deletions within or close to an essential domain, it is possible that these mutations also exert a dominant-negative or gain-of-function effect, but this has still to be studied. Until now, mutations in *SMARCB1* have been described in 13 SSRIDD patients. These are four different heterozygous missense mutations between nucleotides 1089 and 1130 (p.Lys363Asn, p.Arg366Cys, p.Arg374Gln, p.Arg377His) and one recurrent in-frame deletion at nucleotides 1091_1093 (p.Lys364del) (Tsurusaki et al., 2012, 2014b; Santen et al., 2013; Wieczorek et al., 2013; Gossai et al., 2015). These 14 *SMARCB1* mutations are all located within the C-terminus of the protein, close to the SNF5 effector domain (**Figure 6C**).

Germline mutations in *SMARCB1* can also cause different tumor predisposition syndromes. Germline whole-gene or partial gene deletions, as well as truncating mutations of *SMARCB1* cause a cancer predisposition syndrome called Rhabdoid Tumor Predisposition Syndrome 1 (RTPS1; OMIM 609322). Thus, further supporting the notion that SSRIDD-associated *SMARCB1*-mutations likely act through a dominant-negative or gain-of-function effect. Schwannomatosis (OMIM 162091) as the result of a germline *SMARCB1* missense mutation in combination with the acquisition of an independent *NF2* gene mutation and acquired loss of heterozygosity at the *SMARCB1*-*NF2* locus on chromosome 22 was described by Van den Munckhof et al. (2012) and Gossai et al. (2015). The patient described by Gossai et al., also showed a SSRIDD phenotype. As opposed to the family described by Van den Munckhof et al. (2012), the mutation in this patient was located within exon 9, and thus within the C-terminal SSRIDD-critical region of the gene. Moreover, one previously described mutation in a patient with a Kleefstra syndrome-like phenotype was located near the N-terminus (Kleefstra et al., 2012). These results indicate genotype-phenotype correlations, such that N-terminal missense mutations may cause Kleefstra-like syndrome or predispose to schwannomatosis, while C-terminal missense mutations cause SSRIDD and possibly also a predisposition to schwannomatosis. Further studies are needed to confirm these preliminary genotype-phenotype correlations.

Mutations in *SMARCE1* have been described in six patients with a clinical diagnosis of CSS in the literature. These mutations are all heterozygous missense mutations, located within the HMG box between amino acids 73 and 126 (Tsurusaki et al., 2012; Santen et al., 2013; Wieczorek et al., 2013; Zarate et al., 2016) (**Figure 6D**). All mutations were *de novo*, except for one mutation in an affected mother-offspring pair (Santen et al., 2013). Germline *SMARCE1* loss-of-function mutations are found in patients with a hereditary predisposition to spinal and cranial clear cell meningiomas (Smith et al., 2013, 2014), again indicating a different pathomechanism in SSRIDD, in analogy to the other SSRIDD genes.

### Mutations in *SOX11*

**FIGURE 7:**
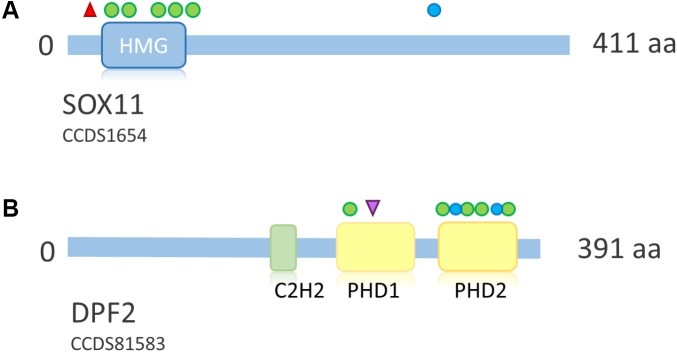
Overview of *SOX11* and *DPF2* protein structure and distribution of mutations across the proteins. **(A)** Schematic representation of *SOX11*. Point mutations according to Tsurusaki et al. (2014a), Hempel et al. (2016), Okamoto et al. (2017), and Khan et al. (2018). Protein structure according to uniprot ID P35716. Deletions not shown due to their size. **(B)** Schematic representation of DPF2. Mutations according to Vasileiou et al. (2018). Protein structure according to uniprot ID Q92785. Blue circle, frameshift mutation; red triangle, stop mutation; green circle, missense mutation; purple triangle, splice site mutation. HMG, high mobility group box; C2H2, zinc finger C2H2-type; PHD, plant homeo domain.

SOX11 is a transcription factor downstream of SWI/SNF. Its expression is regulated by the Smarca4 containing Pax6–BAF complex and leads to expression of growth differentiation factor 5 (GDF5), a TGF-beta superfamily member (Ninkovic et al., 2013). Tsurusaki et al. (2014a) first described *de novo* heterozygous missense mutations within the HMG box of *SOX11* in two patients with a SSRIDD phenotype which overlaps CSS. Hempel et al. (2016) described three point mutations (one stop and two missense mutations) and seven large deletions of *SOX11*, encompassing *SOX11* and, in some cases, also neighboring genes. Okamoto et al. (2017) described one patient with a missense mutation, and Khan et al. (2018) described a patient with a C-terminal frameshift mutation in *SOX11* (**Figure 7A**). The descriptions of two truncating mutations and seven heterozygous deletions of *SOX11* indicate that haploinsufficiency is the underlying mechanism for these mutations. However, the pLI of this gene is 0.34, due to one LoF mutation in the ExAC dataset, which was eligible for analysis. In fact, the ExAC browser shows a stop mutation at amino acid position 11 and a frameshift at position 306. Assuming that these variants are not artifacts, this finding either indicates a different mechanism to be considered or reduced penetrance of *SOX11* mutations. The missense constraint for this gene is 4.25 and the HI index is borderline with 20.02% (**Table 2**). These metrics might also be influenced by the size and structure of the gene (one large exon, only partially coding). Neirijnck et al. (2018) described missense variants in *SOX11* in patients with congenital anomalies of the kidney and urinary tract. However, since some of the described variants were observed in the ExAC browser and segregation analysis was not performed, the results are inconclusive. The authors also discuss reduced penetrance of *SOX11* variants. More studies are needed in order to confirm the full spectrum and the pathomechanism of mutations in *SOX11*.

Interestingly, recessive and dominant mutations in *GDF5* (OMIM 601146), the downstream target of SOX11, cause a variety of skeletal phenotypes, in particular different forms of brachydactyly. Possibly, dysregulation of GDF5 as a result of a SWI/SNF mutation might play a role in the development of the skeletal manifestations of CSS.

### Mutations in *DPF2*

Heterozygous *de novo* mutations in *DPF2* were recently described by Vasileiou et al. (2018). They identified five missense mutations, one splice-site mutation, one frameshift mutation and one splice-site mutation leading to a frameshift on protein level in eight individuals with a CSS-like phenotype. The detected mutations cluster in the two PHD finger domains (**Figure 7B**). DPF2 is a subunit of the BAF complex, but not of the PBAF complex (Hodges et al., 2016). *DPF2* has a pLI of 1.0 and no truncating variants are annotated in the ExAC browser. An HI index of 10.25 indicates haploinsufficiency and a *Z* score of 3.3 indicates missense constraint (**Table 2**). These metrics, together with the finding of truncating mutations, indicate haploinsufficiency as the pathomechanism. However, since there are two homologs, DPF1 and DPF3, which might have redundant functions, a dominant-negative effect of mutations in DPF2 is also plausible. Based on functional studies in patient cell lines, Vasileiou et al. (2018) suggested that the identified missense mutations probably disrupt the tandem PHD finger’s functional cohesion and capacity to recognize H3 histone modifications. Imaging studies showed sequestration of SMARCA4 and wild-type DPF2 into nuclear aggregations in mutant cells, suggesting a disruption of the physiological protein interactions in the BAF complex due to a dominant-negative effect of the *DPF2* missense mutations (Vasileiou et al., 2018).

## Phenotypic Diversity and Modifying Factors Within the SSRIDD Spectrum

The immense phenotypic variety among patients with SWI/SNF mutations, especially in those with *ARID1B* mutations, prompts the question, which might be the modifying factors. The fact that mutations in human SWI/SNF-related disorders are dominant implies that the ATP-dependent chromatin remodeling machinery is dosage sensitive (Kadoch et al., 2017). For instance, mutations in the ATPase domain of SMARCA4 abolish the interaction with and, thereby, the eviction of PRC1 from the nucleosome (Stanton et al., 2017), leading to genomewide polycomb accumulation (Hodges et al., 2018), higher PRC1 occupancy at the nucleosome and transcriptional silencing (Stanton et al., 2017). Since, the interaction between the Trx and PcG complexes is a dynamic system, many factors might additionally influence the individual effect of a mutation in any subunit: single nucleotide polymorphisms or private variants in other SWI/SNF subunit genes, at enhancer sites or in transcription factors might tip off the balance of expression/repression in one or the other direction. Depending on the modifying factor, this might have unspecific effects such as an influence on the degree of ID or possibly even quite specific effects such as the presence or absence of the CSS cardinal feature, fifth-finger hypoplasia.

Phenotypic expression might also be influenced by mosaicism, as observed in patients with mutations in *ARID1A* (Santen et al., 2013; Wieczorek et al., 2013). With the notion of a dosage-sensitive system in mind and taking into account the specialized functions and subunit composition of the SWI/SNF complexes in different tissues (Kadoch et al., 2017), it makes sense to postulate that the allele frequency of a mutated allele would influence disease expression. Possibly, this mechanism might also play a role for *ARID1B*-associated SSRIDD. Although no mosaic mutations have yet been described for *ARID1B*, experience from other disorders of chromatin dysregulation, such as Cornelia-de-Lange syndrome, shows that mosaic mutations are more common than previously thought and are best detected by high coverage NGS panels (Ansari et al., 2014). In patients with a strong clinical suspicion of SSRIDD that test negative for mutations in the known genes, a mosaic mutation that is absent from blood lymphocyte DNA due to revertant mosaicism may be worth considering. Deep sequencing of DNA from other tissues, such as buccal mucosa, would be the investigation of choice to answer this question. Further studies are needed to identify the true rate of mosaicism in SSRIDD.

## Conclusion

Since the SWI/SNF complex is of prime clinical importance both in human genetics as well as in oncology, vivid, and elaborate research is being conducted worldwide in order to unravel the cellular and developmental effects of altered chromatin regulation, and to understand the pathogenesis of SWI/SNF related disorders. The details of the functional research conducted so far are beyond the scope of this review, which is focused on the clinical and molecular genetic aspects of SSRIDD.

The phenotypes caused by mutations in the different SWI/SNF genes merge into the same clinical spectrum defined by ID, speech delay, behavioral problems, ectodermal, and skeletal features, as well as similar facial features. Although there are clinical features that are more common in one entity or the other, for example the swelling of small joints as one of the cardinal features of NCBRS, it appears that there is not a single feature that is exclusively present in one disorder. We have emphasized in this review that, although classic NCBRS is a well recognizable phenotype, some of the atypical forms of CSS caused by mutations in *SMARCB1, SMARCE1* as well as *ARID1A* and *ARID2* may strongly resemble NCBRS. We are therefore convinced that SSRIDD is a clinical continuum that contains ID, CSS, and NCBRS. In the era of NGS, it becomes more and more obvious that it is the clinical geneticist’s task to identify a disease spectrum in order to choose the appropriate gene panel and then to link the molecular data to an individual’s phenotype. In this respect, it is important to know the full clinical spectrum of SSRIDD to also identify mild and atypical cases.

The expressivity of SSRIDD might be influenced by modifying variants in other related genes or by mosaicism, leading to a dosage effect. Targeted NGS panels are advantageous for the detection of mosaic mutations due to higher attainable coverage compared with WES. Retesting with DNA from a tissue other than blood (i.e., buccal swap) may be considered in patients with a strong clinical suspicion of SSRIDD who tested negative for mutations in the known genes.

Many of the cases from the initial studies of 2012 and 2013 that remained without a genetic diagnosis have now been solved through WES (Tsurusaki et al., 2014a; Bramswig et al., 2015, 2017), either by the identification of differential diagnoses or of new causative SSRIDD genes such as *SOX11* and *ARID2*. Since there remain unsolved cases, we expect that the contribution of other SWI/SNF-associated genes to the heterogeneous spectrum of SSRIDD will be reported in the near future.

## Web Resources

DECIPHER (https://decipher.sanger.ac.uk); Exome Aggregation Consortium (ExAC), Cambridge, MA (URL: http://exac.broadins titute.org); Exome Variant Server, NHLBI GO Exome Sequencing Project (ESP), Seattle, WA (http://evs.gs.washington.edu/EVS/); Online Mendelian Inheritance in Man (OMIM^®^. McKusick-Nathans Institute of Genetic Medicine, Johns Hopkins University, Baltimore, MD (http://omim.org/); PubMed (https://www.ncbi.nlm.nih.gov/pubmed); and Uniprot (www.uniprot.org).

## Author Contributions

BW and NB conceptualized the manuscript content. NB wrote the manuscript. BW reviewed the manuscript for important intellectual content.

## Conflict of Interest Statement

The authors declare that the research was conducted in the absence of any commercial or financial relationships that could be construed as a potential conflict of interest.
