# Supplementary material for: Mutational Landscapes and Phenotypic Spectrum of SWI/SNF-Related Intellectual Disability Disorders
Source: Front Mol Neurosci. 2018 Aug 3;11:252. doi: 10.3389/fnmol.2018.00252 (PMC6085491; doi:10.3389/fnmol.2018.00252)
Supplement: Supplementary file 1 [file Data_Sheet_1.DOCX]

**Supplementary Table 1. Animal models of SWI/SNF relevant to SSRIDD**

| **Gene** | **Species** | **Major finding** | **Reference** |
| --- | --- | --- | --- |
| *ARID1A* | Mouse | Arid1a-DNA interactions are required for promoter occupancy by SWI/SNF. | Chandler, R. L., et al. (2013). |
|  | Mouse | The SWI/SNF BAF-A complex is essential for neural crest development. | Chandler, R. L., and Magnuson, T. (2016). |
|  | Mouse | Arid1a and Arid1b are expressed at different timepoints during the cell cycle, indicating differential patterns of incorporation to the complex as means of modulation of SWI/SNF activity during proliferation and arrest. | Flores-Alcantar, A., et al. (2011). |
|  | Mouse | ES cell pluripotency and germ-layer formation require Arid1a. | Gao, X., et al. (2008). |
|  | Mouse | Arid1a is essential for normal cell cycle arrest. | Nagl, N. G., et al. (2005). |
| *ARID1B* | Mouse | Arid1b haploinsufficient mice reveal neuropsychiatric phenotypes and growth impairment responsive to growth hormone therapy. | Celen, C., et al. (2017). |
|  | Mouse | Arid1a and Arid1b are expressed at different timepoints during the cell cycle, indicating differential patterns of incorporation to the complex as means of modulation of SWI/SNF activity during proliferation and arrest. | Flores-Alcantar, A., et al. (2011). |
|  | Mouse | Arid1b haploinsufficiency disturbs interneuron development. *Arid1b*-heterozygous mice exhibit abnormal cognitive and social behaviors, responsive to treatment with a GABA_A_ receptor modulator. | Jung, E.-M., et al. (2017). |
|  | Mouse | Arid1b plays an essential role in Dendritic Arborization and Spine Morphology of Developing Pyramidal Neurons. | Ka, M., (2016). |
|  | Mouse | *Arid1b* haploinsufficiency causes ASD-like phenotypes in mice. | Shibutani, M., et al. (2017). |
| *ARID2* | Mouse | Arid2 is required for heart morphogenesis and coronary artery development. | He, L., et al. (2014). |
|  | Mouse | ARID2-containing complexes play a major role in lineage commitment and differentiation. | Xu, F., et al. (2012). |
| *SMARCA4* | Mouse | Dual active/repressive function of Smarca4 at distal regulatory sequences in vivo and support of its role in tissue-specific gene regulation during embryonic development. | Attanasio, C., et al. (2014). |
|  | Mouse | Smarca4-dependent chromatin remodelling is not essentially required during oligodendroglial differentiation. | Bischof, M., et al. (2015). |
|  | Mouse | Smarca4 plays an essential role in primitive erythropoiesis and vascular development. | Griffin, C. T., et al. (2008). |
|  | Mouse | Smarca4 maintains cardiomyocytes in an embryonic state, and interacts with other chromatin-modifying factors to control developmental and pathological gene expression. | Hang, C. T., et al. (2010). |
|  | Mouse | Smarca4 is required for mouse lens fiber cell terminal differentiation and its denucleation. | He, S., et al. (2010). |
|  | Mouse | Smarca4 has an essential role in spermatogenesis, and in programmed recombination and repair events during meiosis. | Kim, Y., et al. (2012). |
|  | Mouse | Smarca4 interacts with SOX10 to establish the melanocyte lineage and to promote differentiation. | Marathe, H. G., et al. (2017). |
|  | Mouse | p63 and Smarca4 play an essential role in remodelling the higher-order chromatin structure of the EDC within the 3D nuclear space, as required for the efficient expression of EDC genes in epidermal progenitor cells during skin development. | Mardaryev, A. N., et al. (2014). |
|  | Mouse | Deletion of Smarca4 in cerebellar granule cell precursors causes severe ataxia and motor coordination deficits, and a hypoplastic cerebellum. The deletion also caused an enhanced activity of Wnt/β-catenin signaling. | Moreno, N., et al. (2014). |
|  | Mouse | Smarca4 enables rapid embryonic growth by suppressing genes that regulate cell growth arrest. | Singh, A. P., et al. (2016). |
|  | Mouse | Smarca4 ATPase is required for direct eviction of PRC1 from chromatin. | Stanton, B. Z., et al. (2017). |
|  | Mouse | Smarca4 is required for synapse development and remodeling. | Zhang, Z., et al. (2016). |
|  | Zebrafish | Smarca4 is involved in neurogenesis and neural crest induction in zebrafish. | Eroglu, B., et al. (2006). |
|  | Zebrafish | Smarca4 is involved in retinal cell differentiation in zebrafish. | Gregg, R. G., et al. (2003). |
|  | Zebrafish | Smarca4 is involved in the differentiation of skeletogenic neural crest in zebrafish. | Lou, X., et al. (2015). |
|  | Zebrafish | Sox11 is required to maintain proper levels of Hedgehog signaling during vertebrate ocular morphogenesis. | Pillai-Kastoori, L., et al. (2014). |
|  | Zebrafish | Interactions between Brg1 and transcription factors is a dosage sensitive system possibly explaining TF haploinsufficiency in CHD. | Takeuchi, J. K., et al. (2011). |
|  | Zebrafish | Smarca4 mutation causes abnormal retinal development and gene expression profile in retina and RPE. | Zhang, L., et al. (2014). |
| *SMARCB1* | Mouse | Smarcb1 is essential for embryonic development and tumor suppression. | Klochendler-Yeivin, A., et al. (2000). |
|  | Mouse | Increased DNA damage sensitivity and apoptosis in cells lacking Smarcb1. | Klochendler-Yeivin, et al. (2006). |
|  | Mouse | Deletion of Smarcb1 in cerebellar granule cell precursors causes severe ataxia and motor coordination deficits, and a hypoplastic cerebellum. The deletion also caused an enhanced activity of Wnt/β-catenin signaling. | Moreno, N., et al. (2014). |
| *SOX11* | Mouse | Transcription factors SOX4 and SOX11 function redundantly to regulate the development of mouse retinal ganglion cells. Deletion of both factors abolishes RGC development. | Jiang, Y., et al. (2013). |
|  | Mouse | Sox11 is required to maintain proper levels of Hedgehog signaling during vertebrate ocular morphogenesis. | Pillai-Kastoori, L., et al. (2014). |
|  | Zebrafish | Sox11 enhances early osteoblast differentiation by facilitating proliferation and the survival of mesenchymal and osteoblast progenitors. | Gadi, J., et al. (2013). |
|  | Zebrafish | Sox11 regulates vascular development in zebrafish. | Schmitt, C. E., et al. (2013). |
|  | Zebrafish | SOX11 mutant zebrafish show brain abnormalities. | Tsurusaki, Y., et al. (2014). |
| Abbreviations: ASD = autism spectrum disorder, EDC = epidermal differentiation complex, TF = transcription factor, CHD = congenital heart disease, RGC = retinal ganglion cells, RPE = retinal pigment epithelium. | | | |

**Supplementary Table 2. Pathogenic or likely pathogenic *ARID1B* point mutations extracted from LOVD.**

| **Exon** | **cDNA** | **Type** | **Protein** | **Reference** | **De novo** | Patients (n =) |
| --- | --- | --- | --- | --- | --- | --- |
| 1 | c.64_74del | f | p.(Glu22Glnfs*206) | - | De novo | 1 |
| 1 | c.1114dup | f | p.(Arg372Profs*163) | Hoyer et al 2012 | De novo | 1 |
| 1 | c.1222dup | f | p.(Gln408Profs*127) | - | De novo | 1 |
| 1 | c.1235dup | f | p.(Ser413Valfs*122) | - | De novo | 1 |
| 1 | c.1259del | f | p.(Asn420Ilefs*10) | - | De novo | 1 |
| 1 | c.1259dup | f | p.(Asn420Lysfs*115) | - | De novo | 2 |
| 1 | c.1346del | f | p.(Pro449Argfs*53) | - | De novo | 1 |
| 1 | c.1389_1398del | f | p.(Ala464Serfs*35) | - | De novo | 1 |
| 1 | c.1392_1402del | f | p.(Gln467Argfs*64) | - | De novo | 1 |
| 1 | c.1439del | f | p.(Gly480Alafs*22) | - | De novo | 1 |
| 1 | c.1468_1472del | f | p.(Trp490Glyfs*43) | - | De novo | 1 |
| 1 | c.1540dup | f | p.(Gln514Profs*21) | Wieczorek 2013 | De novo | 1 |
| 2 | c.1584del | f | p.(Leu528Phefs*65) | - | Unknown | 1 |
| 2 | c.1595del | f | p.(Gly532Alafs*61) | - | Unknown | 1 |
| 2 | c.1678_1688del | f | p.(Ile560Glyfs*89) | Tsurusaki 2012 | De novo | 1 |
| 2 | c.1713del | f | p.(Gly572Glufs*21) | - | De novo | 1 |
| 4 | c.1808dup | f | p.(Ser603Argfs*50) | Wieczorek 2013 | De novo | 1 |
| 4 | c.1871del | f | p.(Pro624Hisfs*44) | Wieczorek 2013 | De novo | 1 |
| 5 | c.1996_2003dup | f | p.(Asn668Lysfs*3) | - | Unknown | 1 |
| 6 | c.2062del | f | p.(Leu688Serfs*9) | - | De novo | 1 |
| 6 | c.2072_2083delinsAGGGAGGT | f | p.(Gly691Glufs*5) | - | Unknown | 1 |
| 7 | c.2306_2308delinsTCCGCAGCCACTCC | f | p.(Pro769Leufs*17) | - | De novo | 1 |
| 8 | c.2541_2542insG | f | p.(Pro849Thrfs*4) | - | De novo | 1 |
| 9 | c.2598del | f | p.(Tyr867Thrfs*47) | - | Unknown | 1 |
| 9 | c.2625dup | f | p.(Ile876Tyrfs*66) | - | De novo | 1 |
| 9 | c.2723del | f | p.(Pro908Hisfs*6) | Wieczorek 2013 | De novo | 1 |
| 9 | c.2803dup | f | p.(Met935Asnfs*7) | - | Unknown | 1 |
| 9 | c.2877del | f | p.(Ser959Argfs*9) | - | De novo | 1 |
| 10 | c.2891_2892insAC | f | p.(Phe964Leufs*5) | - | De novo | 1 |
| 10 | c.2998del | f | p.(Ala1000Argfs*5) | - | De novo | 1 |
| 12 | c.3208_3209del | f | p.(Lys1070Alafs*47) | - | De novo | 1 |
| 12 | c.3323_3324del | f | p.(Lys1108Argfs*9) | Hoyer et al 2012 | De novo | 1 |
| 13 | c.3394_3395insTA | f | p.(Gly1132Valfs*11) | - | De novo | 1 |
| 14 | c.3586dup | f | p.(Gln1196Profs*14) | Wieczorek 2013 | De novo | 2 |
| 15 | c.3843del | f | p.(Phe1282Leufs*4) | - | De novo | 1 |
| 15 | c.3846dup | f | p.(Gly1283Trpfs*38) | - | De novo | 1 |
| 18 | c.4143_4148delinsG | f | p.(Pro1382Serfs*75) | DDDS 2015 | De novo | 3 |
| 18 | c.4216_4217insTGCTGCTGCTCCTACTCGG | f | p.(Gln1406Leufs*59) | Wieczorek 2013 | De novo | 1 |
| 18 | c.4273dup | f | p.(Tyr1425Leufs*34) | - | De novo | 1 |
| 18 | c.4619_4628del | f | p.(Gln1541Argfs*35) | Santen et al 2012 | De novo | 1 |
| 18 | c.4770_4771delinsG | f | p.(Gln1591Argfs*23) | Wieczorek 2013 | De novo | 1 |
| 18 | c.4820_4825delinsAGGCT | f | p.(Thr1607Lysfs*7) | - | De novo | 1 |
| 18 | c.4821del | f | p.(Pro1609Leufs*5) | - | De novo | 1 |
| 19 | c.4911_4915del | f | p.(Trp1637Cysfs*6) | - | De novo | 1 |
| 19 | c.4916_4917del | f | p.(Val1639Aspfs*5) | - | De novo | 1 |
| 20 | c.5264_5270delinsCAG | f | p.(Glu1756Alafs*9) | DDDS 2015 | De novo | 1 |
| 20 | c.5267_5270del | f | p.(Glu1756Alafs*9) | - | De novo | 1 |
| 20 | c.5394_5397del | f | p.(Phe1798Leufs*52) | - | De novo | 1 |
| 20 | c.5547dup | f | p.(Ser1851Lysfs*5) | Trujillano 2017 | De novo | 1 |
| 20 | c.5570_5573del | f | p.(Lys1857Serfs*17) | Trujillano 2017 | De novo | 1 |
| 20 | c.5632del | f | p.(Asp1878Metfs*96) | Tsurusaki 2012 | Unknown | 2 |
| 20 | c.5635del | f | p.(Asp1879Thrfs*95) | - | De novo | 1 |
| 20 | c.5789delC | f | p.(Pro1930Leufs*44) | - | De novo | 1 |
| 20 | c.6233del | f | p.(Pro2078Leufs*21) | - | De novo | 1 |
| 20 | c.6439dup | f | p.(Arg2147Lysfs*45) | Wieczorek 2013 | De novo | 1 |
| 20 | c.6463_6473del | f | p.(Ser2155Leufs*33) | Hoyer et al 2012 | De novo | 1 |
| 20 | c.6560del (published as c.6559delCTinsC) | f | p.(Leu2187Argfs*6) | DDDS 2015 | De novo | 1 |
| 18 | c.4346G>C | m | p.(Gly1449Ala) | - | De novo | 1 |
| 20 | c.5998G>T | m | p.(Asp2000Tyr) | - | De novo | 1 |
| 20 | c.6092T>C | m | p.(Ile2031Thr) | - | Unknown | 1 |
| 2 | c.1621C>T | s | p.(Gln541*) | DDDS 2015 | De novo | 1 |
| 2 | c.1729C>T | s | p.(Gln577*) | DDDS 2015 | De novo | 1 |
| 4 | c.1903C>T | s | p.(Gln635*) | Tsurusaki 2012 | De novo | 1 |
| 4 | c.1914C>A | s | p.(Tyr638*) | DDDS 2015 | De novo | 1 |
| 6 | c.2248C>T | s | p.(Arg750*) | Wieczorek 2013 | De novo | 1 |
| 8 | c.2507C>G | s | p.(Ser836*) | DDDS 2015 | De novo | 2 |
| 9 | c.2692C>T | s | p.(Arg898*) | Wieczorek 2013 | De novo | 1 |
| 10 | c.2941C>T | s | p.(Gln981*) | - | De novo | 1 |
| 12 | c.3223C>T | s | p.(Arg1075*) | Santen et al 2012 | De novo | 1 |
| 12 | c.3304C>T | s | p.(Arg1102*) | Tsurusaki 2012, Hoyer et al 2012 | De novo | 2 |
| 12 | c.3430C>T | s | p.(Gln1144*) | Wieczorek 2013 | De novo | 2 |
| 13 | c.3481G>T | s | p.(Glu1161*) | - | De novo | 1 |
| 16 | c.3919C>T | s | p.(Gln1307*) | Hoyer et al 2012 | De novo | 1 |
| 16 | c.4009C>T | s | p.(Arg1337*) | - | De novo | 1 |
| 17 | c.4038T>A | s | p.(Tyr1346*) | Hoyer et al 2012 | De novo | 2 |
| 17 | c.4045C>T | s | p.(Gln1349*) | DDDS 2015 | De novo | 1 |
| 17 | c.4098C>G | s | p.(Tyr1366*) | - | De novo | 1 |
| 18 | c.4357C>T | s | p.(Gln1453*) | - | De novo | 1 |
| 18 | c.4620C>A | s | p.(Tyr1540*) | - | Unknown | 3 |
| 19 | c.4911G>A | s | p.(Trp1637*) | - | De novo | 1 |
| 20 | c.5071del | s | p.(Leu1691*) | Wieczorek 2013 | De novo | 1 |
| 20 | c.5103T>A | s | p.(Tyr1701*) | - | De novo | 1 |
| 20 | c.5329A>T | s | p.(Lys1777*) | Santen et al 2012 | De novo | 1 |
| 20 | c.5404C>T | s | p.(Arg1802*) | DDDS 2015, | De novo | 1 |
| 20 | c.5457G>A | s | p.(Trp1819*) | Wieczorek 2013 | De novo | 2 |
| 20 | c.5458C>T | s | p.(Gln1820*) | - | De novo | 1 |
| 20 | c.5623_5625delinsTGACGTCT | s | p.(Ala1875*) | - | Unknown | 1 |
| 20 | c.5776C>T | s | p.(Arg1926*) | DDDS 2015 | De novo | 1 |
| 20 | c.5968C>T | s | p.(Arg1990*) | - | De novo | 5 |
| 20 | c.6038G>A | s | p.(Trp2013*) | - | De novo | 4 |
| 20 | c.6041G>A | s | p.(Trp2014*) | Wieczorek 2013 | De novo | 1 |
| 20 | c.6120C>G | s | p.(Tyr2040*) | - | Unknown | 2 |
| 20 | c.6382C>T | s | p.(Arg2128*) | Wieczorek 2013 | De novo | 1 |
| 20 | c.6516C>G | s | p.(Tyr2172*) | - | De novo | 2 |
| 1i | c.1542+1G>A | spl | - | - | Unknown | 1 |
| 17 | c.4110G>A | spl | p.His1339Ilefs*77 | Hoyer et al 2012 | De novo | 1 |
| 11i | c.3135+1G>C | spl | - | - | Unknown | 1 |
| 18i | c.4895-1G>A | spl | - | DDDS 2015 | De novo | 1 |
| Del = deletion, dup = duplication, f = frameshift mutation, s = stop mutation, m = missense mutation, spl = splice site mutation. NM_020732.3. | | | | | | |

**Supplementary Figure 1. Pie-chart of mutation types in *ARID1B* extracted from LOVD database*.***

**Supplementary References**

Attanasio, C., Nord, A. S., Zhu, Y., Blow, M. J., Biddie, S. C., Mendenhall, E. M., et al. (2014). Tissue-specific SMARCA4 binding at active and repressed regulatory elements during embryogenesis. *Genome Res.* 24, 920–929. doi:[10.1101/gr.168930.113](https://doi.org/10.1101/gr.168930.113).

Bischof, M., Weider, M., Küspert, M., Nave, K.-A., and Wegner, M. (2015). Brg1-dependent chromatin remodelling is not essentially required during oligodendroglial differentiation. *J. Neurosci.* 35, 21–35. doi:[10.1523/JNEUROSCI.1468-14.2015](https://doi.org/10.1523/JNEUROSCI.1468-14.2015).

Celen, C., Chuang, J.-C., Luo, X., Nijem, N., Walker, A. K., Chen, F., et al. (2017). Arid1b haploinsufficient mice reveal neuropsychiatric phenotypes and reversible causes of growth impairment. *Elife* 6. doi:[10.7554/eLife.25730](https://doi.org/10.7554/eLife.25730).

Chandler, R. L., Brennan, J., Schisler, J. C., Serber, D., Patterson, C., and Magnuson, T. (2013). ARID1a-DNA interactions are required for promoter occupancy by SWI/SNF. *Mol. Cell. Biol.* 33, 265–280. doi:[10.1128/MCB.01008-12](https://doi.org/10.1128/MCB.01008-12).

Chandler, R. L., and Magnuson, T. (2016). The SWI/SNF BAF-A complex is essential for neural crest development. *Dev. Biol.* 411, 15–24. doi:[10.1016/j.ydbio.2016.01.015](https://doi.org/10.1016/j.ydbio.2016.01.015).

Eroglu, B., Wang, G., Tu, N., Sun, X., and Mivechi, N. F. (2006). Critical role of Brg1 member of the SWI/SNF chromatin remodeling complex during neurogenesis and neural crest induction in zebrafish. *Dev. Dyn.* 235, 2722–2735. doi:[10.1002/dvdy.20911](https://doi.org/10.1002/dvdy.20911).

Flores-Alcantar, A., Gonzalez-Sandoval, A., Escalante-Alcalde, D., and Lomelí, H. (2011). Dynamics of expression of ARID1A and ARID1B subunits in mouse embryos and in cells during the cell cycle. *Cell Tissue Res.* 345, 137–148. doi:[10.1007/s00441-011-1182-x](https://doi.org/10.1007/s00441-011-1182-x).

Gadi, J., Jung, S.-H., Lee, M.-J., Jami, A., Ruthala, K., Kim, K.-M., et al. (2013). The transcription factor protein Sox11 enhances early osteoblast differentiation by facilitating proliferation and the survival of mesenchymal and osteoblast progenitors. *J. Biol. Chem.* 288, 25400–25413. doi:[10.1074/jbc.M112.413377](https://doi.org/10.1074/jbc.M112.413377).

Gao, X., Tate, P., Hu, P., Tjian, R., Skarnes, W. C., and Wang, Z. (2008). ES cell pluripotency and germ-layer formation require the SWI/SNF chromatin remodeling component BAF250a. *Proc. Natl. Acad. Sci. U.S.A.* 105, 6656–6661. doi:[10.1073/pnas.0801802105](https://doi.org/10.1073/pnas.0801802105).

Gregg, R. G., Willer, G. B., Fadool, J. M., Dowling, J. E., and Link, B. A. (2003). Positional cloning of the young mutation identifies an essential role for the Brahma chromatin remodeling complex in mediating retinal cell differentiation. *Proc. Natl. Acad. Sci. U.S.A.* 100, 6535–6540. doi:[10.1073/pnas.0631813100](https://doi.org/10.1073/pnas.0631813100).

Griffin, C. T., Brennan, J., and Magnuson, T. (2008). The chromatin-remodeling enzyme BRG1 plays an essential role in primitive erythropoiesis and vascular development. *Development* 135, 493–500. doi:[10.1242/dev.010090](https://doi.org/10.1242/dev.010090).

Hang, C. T., Yang, J., Han, P., Cheng, H.-L., Shang, C., Ashley, E., et al. (2010). Chromatin regulation by Brg1 underlies heart muscle development and disease. *Nature* 466, 62–67. doi:[10.1038/nature09130](https://doi.org/10.1038/nature09130).

He, L., Tian, X., Zhang, H., Hu, T., Huang, X., Zhang, L., et al. (2014). BAF200 is required for heart morphogenesis and coronary artery development. *PLoS ONE* 9, e109493. doi:[10.1371/journal.pone.0109493](https://doi.org/10.1371/journal.pone.0109493).

He, S., Pirity, M. K., Wang, W.-L., Wolf, L., Chauhan, B. K., Cveklova, K., et al. (2010). Chromatin remodeling enzyme Brg1 is required for mouse lens fiber cell terminal differentiation and its denucleation. *Epigenetics Chromatin* 3, 21. doi:[10.1186/1756-8935-3-21](https://doi.org/10.1186/1756-8935-3-21).

Jiang, Y., Ding, Q., Xie, X., Libby, R. T., Lefebvre, V., and Gan, L. (2013). Transcription factors SOX4 and SOX11 function redundantly to regulate the development of mouse retinal ganglion cells. *J. Biol. Chem.* 288, 18429–18438. doi:[10.1074/jbc.M113.478503](https://doi.org/10.1074/jbc.M113.478503).

Jung, E.-M., Moffat, J. J., Liu, J., Dravid, S. M., Gurumurthy, C. B., and Kim, W.-Y. (2017). Arid1b haploinsufficiency disrupts cortical interneuron development and mouse behavior. *Nat. Neurosci.* 20, 1694–1707. doi:[10.1038/s41593-017-0013-0](https://doi.org/10.1038/s41593-017-0013-0).

Ka, M., Chopra, D. A., Dravid, S. M., and Kim, W.-Y. (2016). Essential Roles for ARID1B in Dendritic Arborization and Spine Morphology of Developing Pyramidal Neurons. *J. Neurosci.* 36, 2723–2742. doi:[10.1523/JNEUROSCI.2321-15.2016](https://doi.org/10.1523/JNEUROSCI.2321-15.2016).

Kim, Y., Fedoriw, A. M., and Magnuson, T. (2012). An essential role for a mammalian SWI/SNF chromatin-remodeling complex during male meiosis. *Development* 139, 1133–1140. doi:[10.1242/dev.073478](https://doi.org/10.1242/dev.073478).

Klochendler-Yeivin, A., Fiette, L., Barra, J., Muchardt, C., Babinet, C., and Yaniv, M. (2000). The murine SNF5/INI1 chromatin remodeling factor is essential for embryonic development and tumor suppression. *EMBO Rep.* 1, 500–506. doi:[10.1093/embo-reports/kvd129](https://doi.org/10.1093/embo-reports/kvd129).

Klochendler-Yeivin, A., Picarsky, E., and Yaniv, M. (2006). Increased DNA damage sensitivity and apoptosis in cells lacking the Snf5/Ini1 subunit of the SWI/SNF chromatin remodeling complex. *Mol. Cell. Biol.* 26, 2661–2674. doi:[10.1128/MCB.26.7.2661-2674.2006](https://doi.org/10.1128/MCB.26.7.2661-2674.2006).

Lou, X., Burrows, J. T. A., and Scott, I. C. (2015). Med14 cooperates with brg1 in the differentiation of skeletogenic neural crest. *BMC Dev. Biol.* 15, 41. doi:[10.1186/s12861-015-0090-9](https://doi.org/10.1186/s12861-015-0090-9).

Marathe, H. G., Watkins-Chow, D. E., Weider, M., Hoffmann, A., Mehta, G., Trivedi, A., et al. (2017). BRG1 interacts with SOX10 to establish the melanocyte lineage and to promote differentiation. *Nucleic Acids Res.* 45, 6442–6458. doi:[10.1093/nar/gkx259](https://doi.org/10.1093/nar/gkx259).

Mardaryev, A. N., Gdula, M. R., Yarker, J. L., Emelianov, V. U., Emelianov, V. N., Poterlowicz, K., et al. (2014). p63 and Brg1 control developmentally regulated higher-order chromatin remodelling at the epidermal differentiation complex locus in epidermal progenitor cells. *Development* 141, 101–111. doi:[10.1242/dev.103200](https://doi.org/10.1242/dev.103200).

Moreno, N., Schmidt, C., Ahlfeld, J., Pöschl, J., Dittmar, S., Pfister, S. M., et al. (2014). Loss of Smarc proteins impairs cerebellar development. *J. Neurosci.* 34, 13486–13491. doi:[10.1523/JNEUROSCI.2560-14.2014](https://doi.org/10.1523/JNEUROSCI.2560-14.2014).

Nagl, N. G., Patsialou, A., Haines, D. S., Dallas, P. B., Beck, G. R., and Moran, E. (2005). The p270 (ARID1A/SMARCF1) subunit of mammalian SWI/SNF-related complexes is essential for normal cell cycle arrest. *Cancer Res.* 65, 9236–9244. doi:[10.1158/0008-5472.CAN-05-1225](https://doi.org/10.1158/0008-5472.CAN-05-1225).

Pillai-Kastoori, L., Wen, W., Wilson, S. G., Strachan, E., Lo-Castro, A., Fichera, M., et al. (2014). Sox11 is required to maintain proper levels of Hedgehog signaling during vertebrate ocular morphogenesis. *PLoS Genet.* 10, e1004491. doi:[10.1371/journal.pgen.1004491](https://doi.org/10.1371/journal.pgen.1004491).

Schmitt, C. E., Woolls, M. J., and Jin, S.-W. (2013). Mutant-specific gene expression profiling identifies SRY-related HMG box 11b (SOX11b) as a novel regulator of vascular development in zebrafish. *Mol. Cells* 35, 166–172. doi:[10.1007/s10059-013-2307-8](https://doi.org/10.1007/s10059-013-2307-8).

Shibutani, M., Horii, T., Shoji, H., Morita, S., Kimura, M., Terawaki, N., et al. (2017). Arid1b Haploinsufficiency Causes Abnormal Brain Gene Expression and Autism-Related Behaviors in Mice. *Int J Mol Sci* 18. doi:[10.3390/ijms18091872](https://doi.org/10.3390/ijms18091872).

Singh, A. P., Foley, J. F., Rubino, M., Boyle, M. C., Tandon, A., Shah, R., et al. (2016). Brg1 Enables Rapid Growth of the Early Embryo by Suppressing Genes That Regulate Apoptosis and Cell Growth Arrest. *Mol. Cell. Biol.* 36, 1990–2010. doi:[10.1128/MCB.01101-15](https://doi.org/10.1128/MCB.01101-15).

Stanton, B. Z., Hodges, C., Calarco, J. P., Braun, S. M. G., Ku, W. L., Kadoch, C., et al. (2017). Smarca4 ATPase mutations disrupt direct eviction of PRC1 from chromatin. *Nat. Genet.* 49, 282–288. doi:[10.1038/ng.3735](https://doi.org/10.1038/ng.3735).

Takeuchi, J. K., Lou, X., Alexander, J. M., Sugizaki, H., Delgado-Olguín, P., Holloway, A. K., et al. (2011). Chromatin remodelling complex dosage modulates transcription factor function in heart development. *Nat Commun* 2, 187. doi:[10.1038/ncomms1187](https://doi.org/10.1038/ncomms1187).

Tsurusaki, Y., Koshimizu, E., Ohashi, H., Phadke, S., Kou, I., Shiina, M., et al. (2014). De novo SOX11 mutations cause Coffin-Siris syndrome. *Nat Commun* 5, 4011. doi:[10.1038/ncomms5011](https://doi.org/10.1038/ncomms5011).

Xu, F., Flowers, S., and Moran, E. (2012). Essential role of ARID2 protein-containing SWI/SNF complex in tissue-specific gene expression. *J. Biol. Chem.* 287, 5033–5041. doi:[10.1074/jbc.M111.279968](https://doi.org/10.1074/jbc.M111.279968).

Zhang, L., Ma, P., Collery, R., Trowbridge, S., Zhang, M., Zhong, W., et al. (2014). Expression profiling of the RPE in zebrafish smarca4 mutant revealed altered signals that potentially affect RPE and retinal differentiation. *Mol. Vis.* 20, 56–72.

Zhang, Z., Cao, M., Chang, C.-W., Wang, C., Shi, X., Zhan, X., et al. (2016). Autism-Associated Chromatin Regulator Brg1/SmarcA4 Is Required for Synapse Development and Myocyte Enhancer Factor 2-Mediated Synapse Remodeling. *Mol. Cell. Biol.* 36, 70–83. doi:[10.1128/MCB.00534-15](https://doi.org/10.1128/MCB.00534-15).
